# STEM education centers: catalyzing the improvement of undergraduate STEM education

**DOI:** 10.1186/s40594-018-0143-2

**Published:** 2018-11-12

**Authors:** Deborah L. Carlisle, Gabriela C. Weaver

**Affiliations:** University of Massachusetts Amherst, 140 Hicks Way, Amherst, MA 01003 USA

**Keywords:** STEM education centers, Undergraduate STEM education, Undergraduate STEM reform, STEM education research, STEM center roles, Network of STEM education centers (NSEC)

## Abstract

**Background:**

With the remarkable attention being paid to STEM education nationally, with the growing engagement of universities and colleges in STEM education reform, and with the rise of STEM education centers, SECs, assisting universities as they strive to achieve these reforms, this research provides insight into the roles of six SECs. Through a multi-dimensional cross-site comparison, we provide a lens into the ways in which SECs function on their campuses, illuminating possibilities for those seeking to strengthen undergraduate STEM education.

**Results:**

SECs play an important networking role on their campuses, where they inform and unify institutional efforts, serving to elevate their visibility and importance both internally and externally. Through their scholarship, SECs contribute to the knowledge base and provide funding, which add resources and incentives for the implementation of evidence-based instructional practices (EBIPs) and STEM education research. SECs augment these efforts with the assessment and evaluation of learning outcomes and curricular innovations. Additionally, SECs act as an internal resource for faculty and instructors providing programs and training to foster the application of EBIPs in STEM courses. Several SECs provide the infrastructure for broader impact activities, and act as an external funding resource.

**Conclusions:**

STEM education centers make key contributions to their institutional environments. While the individual roles of these SECs on their campuses are distinctly unique, an in-depth look across six SECs reveals common areas of focus that allow these centers to enhance the undergraduate teaching and learning experience. Our results suggest that the ability of SECs to link STEM education research with teaching and learning initiatives provides a breadth of impact and attention across organizational levels. The analysis describes the ways in which these centers support institutional goals for undergraduate STEM education and relates these to areas of national priority. This research was carried out as part of a broader study, which informs the organizers of NSEC, the network of STEM education centers.

**Electronic supplementary material:**

The online version of this article (10.1186/s40594-018-0143-2) contains supplementary material, which is available to authorized users.

## Background

The remarkable attention being paid to science, technology, engineering, and mathematics, STEM, education nationally has led to growing engagement of universities and colleges in STEM education reform. These efforts have given rise to the proliferation of STEM education centers (SECs) at many higher education institutions. Because SECs have been identified as a locus of educational change on campuses, they are positioned to serve as unique and powerful agents to address the calls for scaling and sustaining educational change (Singer et al. 2012). Yet, while these SECs may be powerful support structures for achieving undergraduate STEM education reform, we currently have only a superficial understanding of their operations. With the majority of SECs established in 2011 or later, this is an opportunistic time to study the mechanisms through which SECs are having an impact at their local institutions. Further, this study improves our understanding of how such efforts contribute to the broader national priorities for STEM education in the USA.

To address the national need for greater numbers of STEM graduates, much emphasis has been placed on K-16 STEM education (PCAST 2012; NSTC 2013). A significant body of research has focused on the challenges facing our educational systems in their efforts to increase the number of students successfully entering and exiting the STEM education system (Seymour 2002; Project Kaleidoscope 2011; Singer et al. 2012). Our focus here pertains to higher education. To meet the need for more STEM graduates, institutions of higher education are focused upon increasing student success and retention in early gateway STEM courses (Bradforth et al. 2015). This has necessitated a wide variety of changes, at the institutional and departmental levels, and at some universities SECs have been important partners. Taken broadly, these changes impact the culture of the learning environment by enhancing the quality of teaching and learning experiences, while also broadening participation and institutional capacity for STEM learning (Boyd and Wesemann 2009).

Efforts to improve the STEM workforce and science-literate citizens have been steady and on-going. These efforts have “drawn increased attention to the quality of undergraduate science and engineering education and how it can be improved” (Singer et al. 2012). Across the nation, there continues to be concern that undergraduate STEM courses are not providing learning experiences that appropriately engage, motivate, and prepare students for careers. Moreover, it appears that these experiences do not allow equal access for all students to aspire to these careers (NSTC 2013; NSTC 2018, National Academies of Sciences, Engineering, and Medicine 2018). Higher education continues to be challenged by the increasingly diverse population it now educates. To offer guidance for teaching and learning within the STEM disciplines, the National Academies put forth the report on discipline-based education research (DBER) (Singer et al. 2012) and recently released the Indicators for Monitoring Undergraduate STEM Education (National Academies of Sciences, Engineering, and Medicine 2018). Here, we focus on the DBER report, as the proliferation of SECs in 2011 spans the time frame this report was released, and many SECs show significant engagement in DBER scholarship. The report findings recommend that faculty adopt evidenced-based instructional practices (EBIPs), shown to promote student learning outcomes (Bransford et al. 1999). Importantly, it underscores the need for STEM faculty to integrate these instructional strategies with their disciplinary content knowledge to facilitate the practical goal of improved learning. To accomplish this, departments and colleges require disciplinary faculty who have developed the necessary pedagogical content knowledge (PCK) to implement and model best practices, within their home departments (Shulman 1986). To meet this need, many institutions have begun to rely on non-tenure stream teaching faculty (Miller et al. 2017). Others have begun to hire one or two DBER faculty in a few amenable STEM departments. Both SECs and Centers for Teaching and Learning (CTLs) play important roles that facilitate faculty learning and implementation of EBIPs (Collaborating at the Centers 2016).

The DBER report was an important step in recognizing and promoting the scholarship of teaching in STEM disciplines. A focus on educational research within STEM disciplines, by disciplinary scholars outside Colleges of Education, or in partnership with Colleges of Education, has not been prominent. As the Association of American Universities reports, there is a “disproportionate emphasis on research productivity” for the promotion and tenure of faculty, which has led to a growing “tension” between research productivity and teaching (Miller et al. 2017). Studies focused on institutional transformation suggest that DBER may serve as a mechanism through which to address this issue, because it engages disciplinary faculty in educational research and influences departmental reform (Weaver et al. 2016). These studies have also linked engagement in DBER research to grass-root efforts that led to the evolution of SECs (Weaver et al. 2016). More broadly, the application of educational research methods, for the assessment of learning outcomes, has been shown to be a key element influencing shifts in faculty practice toward increased use of EBIPs (Marbach-Ad et al. 2015; Shadle et al. 2017). Numerous findings point to the role of educational research in setting the tone for the adoption of enhanced teaching practices (CIRTL.net 2003, Lund et al. 2015).

It is well known that the pace of change in higher education is exceedingly slow. Studies have shown some of this inertia can be attributed to the dynamics created by vertical organization, which sets up a competitive rather than collaborative atmosphere (Keeling et al. 2007). The demand on STEM faculty in postsecondary research institutions necessitates the demonstration of disciplinary excellence through original research that garners funding, scholarly publications, and peer reviewed presentations, all of which “drive verticality” within STEM departments and colleges (Keeling et al. 2007). Some institutional environments can contribute to a culture where colleges, departments, and faculty are concerned with promoting their own internal goals rather than accomplishing broader institutional purposes (Kuh 1996). To date, the majority of studies in higher education have focused upon factors associated with verticality and not on organizational learning and structures that lead to improved progress and sustained change (Hora and Hunter 2014). SECs provide a mid-level structure, which studies suggest facilitate an organization’s ability to be responsive, because they reward and stimulate activity in ways that traditional vertical structures do not (Diefenbach 2013). Our research provides a lens into the ways in which SEC functions promote progress and decrease inertia, emphasizing the importance of integrating such structures, to span the verticality and hence the siloed existence that is often part of the institutional infrastructure of postsecondary institutions (Keeling et al. 2007).

Research in the field of organizational learning often looks to describe the underlying processes of how organizations learn, change, and adapt (Levitt and March 1988). Here, we provide a descriptive analysis of the functions of six SECs, which provides information of center roles that support horizontal and vertical integration. Understanding these areas contributes to organizational learning, by offering insight into the ways in which institutions might best utilize such a structure. As we describe, these centers engage institutions and departments in processes that foster change in undergraduate STEM education, which if sustained could lead to the adaptation of traditional norms. A center structure offers an institution the opportunity to be proactive in addressing challenges related to STEM education adding institution-level efforts to grass-root approaches at the department level, which may at times be more individualized or reactive in nature (Huber 1991).

Our research aims to answer the following research questions to provide a deeper understanding of the roles of STEM Education Centers.

1. What are the primary functions carried out by SECs on their campuses?
2. What research, programmatic, and/or organizational challenges in STEM education are these SECs currently addressing?
3. In what ways are SECs addressing the national priorities in STEM education?

To answer these research questions, this study takes a systems approach as recommended and often utilized to assess STEM education programs (Wasserman 2010; iTest 2013; Elrod and Kezar 2016; Miller et al. 2017). Understanding center functions necessitates an understanding of the relationships between the centers themselves and those they serve. Due to the non-evaluative nature of this research, these relationships were not measured per se, yet nor were they assumed. To understand these relationships and be able to describe them, we explored the perceived value of the center structure through varied perspectives across faculty and administration, to obtain a sense of the value add of each SEC, and to increase the utility of our findings. The systems orientation used here supports the development of contextualized description, which takes into account a full range of perspectives. “A systems orientation suggests that the quality of system relationships not only varies, but varies across perspectives” and the variance is important to understanding center functions as nested within the institution (Wasserman 2010).

To further guide our approach, this study was informed by the Keck/Project Kaleidoscope Systemic Guide to Institutional Change for STEM Education (Elrod and Kezar 2016). This guide highlights the importance of support from both upper and middle administrative leaders, as well as grassroots faculty leaders, for efforts aimed at strengthening undergraduate STEM to be successful. As such, this guide complemented our systems approach, and our investigation into the organizational levels within higher education. Historically, a unified approach is well documented in education, “Across the three waves of reform, two basic lessons have been learned: first, large-scale reform of science education takes time; and second systemic reforms must include both top-down and bottom-up approaches. Research supports those lessons” (Abell and Lederman 2007). While all centers may not be engaged explicitly in reform or change efforts per se, their mission statements seek to strengthen and improve undergraduate STEM education, thereby putting them in a natural role to engage in progressive action at their local institutions. The systems approach taken here also strengthens the implications for organizational learning, by illuminating the ways in which center functions may contribute to the adaptation of departmental priorities, culture, and routines, to improve the success and retention of undergraduates in STEM.

The purpose of this study was to provide a deeper understanding of SEC operations on their campuses, which also serves to inform a broad national survey of these centers. This study is part of an NSF-funded initiative to form a national network of STEM education centers, the Network of STEM Education Centers, NSEC.

## Methods

### Data collection

This study uses a multiple case design to gain understanding of the ways in which these SECs are supporting the improvement of undergraduate STEM education. A purposive sample of SECs was selected to represent a variety of institutional and center types. This sample included SEC’s from three R1 institutions (one private and two public), one R2 public, and two R3 institutions (one private and one public), as described by their Carnegie classification.^1^ Table 1 provides a broad overview of the purposive sample describing center type and structural features. These centers were selected to be representative of the SEC population based upon review of data compiled from prior studies (Riordan 2014) and center websites. SECs were selected from a sample of 124 centers, with profiles at the Science Education Resource Center (SERC) at Carleton College, based upon the following: (a) variation center type shown in Table 1 under center description: university wide, college wide, research only, teaching and learning, education, and diversity; (b) variation in location (e.g., within a college, within a department, or outside departments, see Table 1); (c) mission and vision statements included specific emphasis in undergraduate STEM education; (d) evidence of past and present engagement in undergraduate STEM improvement through grant awards, e.g., NSF: IUSE, STEP, WIDER, and HHMI, and national initiatives, e.g., AAU; and (e) evidence of dissemination of center work through publications, seminars, and institutes. Centers were invited to participate in our study through email communication. All invitees responded favorably and agreed to be part of the study. This research received approval from the Internal Review Board at our university as well as from those institutions engaged in site visits.

**Table 1:** Institutions and SECs: structural features

Institution	Center description	Structural features
Institution A1 R1, Private High selectivity	University wide STEM Center	Reports to: Provost FTE: 6 Location: Central, outside STEM departments Funding: Institutional
Institution B1 R1, Public Moderate selectivity	STEM Education Research Center	Reports to: Deans FTE: 7 Location: College of Education Funding: Institutional & endowment
Institution C1 R1, Public Moderate selectivity	STEM Teaching and Learning Center	Reports to: Dean FTE: 2.5 Location: College of Science Funding: Institutional (recently transitioned from external)
Institution D2 R2, Public Moderate selectivity	University wide STEM Center	Reports to: Deans FTE: 2.5 Location: Math Funding: 75% External; 25% Institutional
Institution E3 R3, Private Moderate selectivity	College-wide STEM Education Center	Reports to: Dean, VPR FTE: 2.5 Location: College of Science Funding: External
Institution F3 R3, Public Moderate selectivity	STEM Education and Diversity Center	Reports to: Provost, VPR FTE: 5 Location: Peripheral, outside STEM depts Funding: 75% External, 25% Institutional

The sample contains a mix of typical cases as well as expert cases. At each site, data was gathered through the use of semi-structured interviews, artifacts, and observations, and took place during the fall of 2016 and the spring of 2017. Although this was a non-evaluative study, individual site visits followed a systems approach to data collection, as frequently used for site evaluations of STEM initiatives (iTEST 2013; Wasserman 2010). At each institution, interviews were conducted across administrative levels during site visits in order to gather multiple perspectives, and improve the validity of our findings. The levels include interview data from (1) the center director(s) and staff; (2) upper administration (provosts, deans, associate provosts, vice presidents of research), and (3) STEM department chairs and STEM faculty (Table 2). This approach provided an informed understanding of the ways in which upper administrators valued the SEC, the ways in which the faculty were engaging with the SEC, as well as the role of the SEC as perceived from within and without. Within each case, qualitative case study methods (Yin 1994; Merriam 2010) were used to address our research questions and gain a deep contextualized understanding of individual SECs.

**Table 2:** Sources of data from individual site visits

Institution	Interviews^a^				Archival documents		Direct observations	
	Center: director/staff	Upper admin	STEM dept. chairs	STEM faculty	Examples	#	Examples	#
A1 Private R1	7	4	6	11	Peer reviewed publications	3	Seminar	2
					Time line	1	Workshop	1
					Annual reports	2		
B1 Public R1	6	4	7	11	Peer reviewed publications	5	Seminar	2
					Time line	1	Meeting	1
					Annual reports	1		
					Grant descrpt	2		
C1 Public R1	2	3	8	10	Peer reviewed publications	3	Seminar	1
					Time line	1		
					Annual reports	1		
D2 Public R2	3	3	4	7	Peer reviewed publications	2	Seminar	1
E3 Private R3	3	4	5	8	Peer reviewed publications	2	Meeting	1
					Time line	1		
					Annual reports	1		
F3 Public R3	7	5	8	10	Peer reviewed publications	3	Workshops	2
					Time line	2		
					Annual reports	2		

^a^Data collection included 1-h, in-person, semi-structured interviews with university personnel in indicated areas. Faculty, staff, and department chair interviews varied between individual meetings, or groups of 3–5 depending on scheduling factors. Leadership interviews such as Center director and administrators were conducted independently

### Analysis

Multi-case sampling was used to build understanding and extend themes across the purposive sample of SECs. “The use of multi-case sampling adds to the validity and generalisability of the findings” (Miles and Huberman 1994) through replication logic (Eisenhardt 1989; Yin 1994). This analysis uses the contextualized understanding of each SEC derived from the individual site visits to identify broad themes and patterns across each of the six SECs, and so discern how these SECs contribute to undergraduate STEM education. Identified areas were mapped to national priority areas, which were based on a synthesis of three documents: the Federal 5-year Strategic Plan (NSTC 2013), the Association of American Universities (AAU) Framework for Systemic Change (2013), and the Association of American Colleges and Universities (AAC&U) CRUSE Sourcebook for Advancing and Funding Undergraduate STEM (2014). Each of these documents describe guidelines to achieve the national priorities through scaffolding processes and pedagogical best practices aimed at improving the quality of teaching and learning, as well as areas that attend to cultural aspects related to contextual and authentic applications of STEM learning to establish relevancy and broaden participation. SEC functions that aligned with the national priorities identified from the synthesis noted above were grouped into two broad categories: (1) improving STEM learning and (2) broadening participation and institutional capacity.

This research was informed by a previous pilot study from the Association of Public Land Grant Universities, during which STEM Education Centers answered a series of survey questions about their structures and functions, which were used to create profiles for individual centers (Riordan 2014). These profiles are now maintained on the Science Education Resource Center (SERC) website at Carleton College. These profiles were reviewed prior to sample selection to develop an understanding of center types and their primary functions, providing background knowledge and informing the development of the coding scheme for cross-center comparisons. Coding categories for cross-center functions are shown in Table 3. Functions were separated into broad categories (services, programs, and educational research) and clear operational definitions were developed “to allow individual(s) to code over time and multiple researchers to think about the same phenomena” (Miles and Huberman 1994). These definitions were utilized to maintain fidelity in the coding process. The primary author coded across individual centers using NVivo© for efficient analysis and organization. At the initial stages of the analysis, a second researcher independently coded 20% of the cross-center data. Codes were compared and inter-rater reliability was found to be 88%. Several weeks in, as analysis was refined, this process was repeated and inter-rater reliability was found to 90%.

**Table 3:** Coding categories and description

Interview data	Functional categories (roles)	Descriptive text coded for center role
Center: Director Staff	1) Services: Provided to faculty, departments, students, and the institution	Any service provided by the center, some examples include: • Assessment and evaluation • Support for EBIP adoption and classroom innovation • Networking • Administrative, e.g., grant management • Consultation • Broader impacts
	2) Programs: Provided to faculty, departments, students, and the institution Increase awareness and develop skills (e.g., professional development activities, learning communities)	Any program provided by the center, some examples include: • Workshops • Seminars • Institutes • Mentoring • Learning communities
	3) Educational research, and assessment/evaluation activities performed by the center, e.g., associated with curricular interventions, and externally funded initiatives	Any aspect of center engagement, some examples include: • Types of educational research carried out • Types of assessment and evaluation activities performed • Collaboration/partnerships: sharing ideas and solving challenges • Consultation • Building partnerships for
Departments: Chairs Faculty	Comments identifying specific areas of engagement with the center; general impressions	• Engagement in any service, program, or research (per above) • Perceived value of engagement
Upper Admin: Provost Deans VPR	Comments related to center role; value of center; areas of impact; observations of faculty, department, and student engagement; general impressions	Perceived role of the center and value of • For faculty/departments • For students • For the institution

### Coding process

Data were coded to categories and levels using NVivo© in a three-part process to answer our first two research questions. During the initial coding process, interviews with the SEC director(s) and staff were coded to the three functional categories shown in Table 3. Interview data was augmented with artifact and website data describing center functions. Faculty interviews were then coded to these categories to corroborate engagement with these center functions. Likewise, upper administrative interview comments were coded to “the role of the center,” aggregating comments describing the perceived role of the center and the value of center functions in the areas outlined in Table 3. Following this initial process, center functions with faculty and departmental engagement were identified and linked to administrative comments, both specific and broad, addressing these same areas. Properties and dimensions were developed for each of these areas (services, programs, research) from the coded data to obtain a rich contextualized description for individual SECs, which was used to identify themes and patterns across cases.

To answer our third research question of how these functions map onto national priority areas, center functions were selectively coded into two broad categories: improving STEM learning or broadening participation and institutional capacity. These categories were aligned with the identified national and institutional priorities (NSTC 2013; AAU 2013; AAC&U 2014). These broad categories are further detailed and described in Tables 4 and 7 of the “Results” section.

**Table 4:** SEC functions directed toward the improvement of STEM learning

Function	Institution/Center					
	A1	B1	C1	D2	E3	F3
1) Educational research:						
• Conduct, catalyze, consult in STEM education research, e.g., DBER, SoTL, action	x	x	x	x	x	x
• Engage faculty by						
o Leveraging external funding source	x	x	x	x	x	x
o Providing seed funding to initiate/catalyze	x	x	x			x
• Measure outcomes of curricular innovations	x	x	x	x	x	x
• Partner with institutional research to explore base-line data for student success in STEM courses and pathways	x		x		x	x
2) Enhancing the quality of teaching and learning:						
• Develop innovative curricula		x	x		x	
• Affiliated faculty model the use of best practices in home departments	x	x	x	x	x	x
• Disseminate the results of successful studies through seminars, workshops, institutes	x	x	x	x	x	x
• Professional development for faculty who mentor STEM undergraduates in research experiences	x		x	x	x	x
• Research/programs/services aimed at increasing understanding and use of EBIPs	x	x	x		x	x
• Training of learning assistants, teaching assistants, graduate students	x		x		x	x
• Research/programs/services aimed at increasing understanding and use of pedagogies that support diversity and inclusion	x	x	x	x	x	x

(x represents triangulated evidence across academic levels, and in three or more interviews within levels, at individual institutions)

As individual centers were compared, cross-case “searching tactics” (Eisenhardt 1989, p. 541) were used to ensure consistency of analyses and to move beyond initial impressions in order to improve comparative interpretation. Our tactics included juxtaposition of similar cases to illuminate underlying differences, as well as the use of tables and flow charts to summarize and compare between cases. Comparative methods utilized a structured lens to capture properties and dimensions within cases across the different academic levels, and use those to identify intergroup differences. For example, the degree to which these SECs were research focused vs. service/program focused; the degree to which the functions of the SEC supported internal functioning of the institution (e.g., supporting faculty use of EBIP’s) vs. having an external focus (e.g., K-12 outreach); relative size of the SEC (FTE’s). Some of these yielded a small degree of similarity between cases, while others in areas such as educational research practices led to important patterns and greater similarity across cases.

Trustworthiness was established through an extensive audit trail comprised of research memos during the different stages of the analysis process. A collaborating researcher debriefed the primary author approximately every 2 weeks as portions of the analysis were completed and viewed the memos. The audit trail was also used to maintain a record of the details and nuances of the results as they evolved.

## Results

The results are presented in three sections, beginning with a description of the purpose of these SECs, to provide a lens through which to understand their functions. The second section, titled Improving STEM Learning, encompasses two general areas: educational research and enhancing the quality of teaching and learning. The final section, Broadening Participation and Institutional Capacity for STEM Learning, addresses four general areas: building partnerships for research and community for STEM learning, establishing relevance and meeting students’ needs, infrastructure for broader impacts, and support for the K-16 pipeline. The second and third sections were organized to align SEC functions with areas of national priority (NSTC 2013; AAU 2013; AAC&U 2014). This organization allows us to frame the ways in which center functions contribute to research question three. Specific center functions pertaining to each of these areas are outlined in tables to emphasize similarities and differences between each of the six SECs. Throughout each section, a contextualized description was developed using representative examples from primary data sources (i.e., interview comments). As these descriptions were developed, the qualitative data supporting each cross-case finding was based upon triangulated interview comments across our data sources at each institution. The relative occurrence of each finding is documented by section in Additional file 1.

### Purpose and value of center structure

A description of the mission of each center with supporting administrator’s comments describing the value of each SEC, some of which are referenced below, are shown in Additional file 2. At each institution, SECs bring together disaggregated efforts for the purpose of strengthening undergraduate STEM education. In doing so, they bring increased recognition to existing efforts, while also building collaborations to increase the impact and effective use of local resources. “We want to bring these efforts together and give STEM education the importance that it deserves. Every time you create some kind of a structure and you give it a name and you put everybody under that umbrella, then all of a sudden—it is known that it exists” (Dean, Institution E3). The center structure is described across cases as an “umbrella unit,” “clearing-house,” “collaborative,” and “a formal structure to promote synergies.” Importantly, each case describes the significance of this centralized structure as “symbolic of institutional commitment,” across each level. Administrators also describe the center structure as “a mechanism that elevates the institution in the area of STEM education” (Provost, Institution B1). Cross-case data reveal internal as well as external benefits from the increased visibility and status the center structure bestows on institutional efforts.

Much of the SECs’ perceived internal value stems from their contributions to the institutional environment, where they provide incentive and guidance “to infuse active learning strategies and evidence-based practices,” carry out high-quality research to “inform the campus and community at large,” allow the institution to “attract strong leaders,” and increase student opportunities by “funding undergraduate research” as well as other authentic learning experiences. SECs have an important role in providing community for faculty around teaching and educational research. The Associate Dean of the College of Science at Institution C1 describes the significance of this: “We have almost 90 full time professional track faculty in our college. And some of the people who are an n of one in a small department, the Center gives them a community of people who feel the same way about teaching. And we see that we can foster this across departments better than some may think.” SECs are also valued for the central role they play in positioning institutions for external funding, many of which lead to broader impact in areas that engage and support students. Collectively, these contributions allow institutions to take “a fast lane approach” as the Vice President for Research (VPR) at Institution F3 explained, toward the improvement of undergraduate STEM education.

### Improving STEM learning

General center functions directed toward *improving learning* are shown in Table 4. A variety of factors cause SECs to place different emphasis in these areas, including human resources (FTE), financial resources, institutional priorities as influenced by reporting lines, institutional strengths, and the origin of the center. As shown in Table 4, all SECs carry out the functions listed under the first heading of educational research, with one exception: Institutions B1 and D2 do not partner with institutional research to explore base-line data. Under the second heading, Enhancing the Quality of Teaching and Learning, only SECs B1, C1, and E3 develop innovative curricula. For each of these SECs, the research and development component of their mission provides emphasis in this area, and their director’s expertise leads to rich opportunities. While SEC F3 does not have a role in developing innovative curricula, it does partner with the Center for Teaching and Learning to provide assessment expertise for faculty and departments who are implementing innovations. SEC B1 functions exclusively as a research center, and therefore is not engaged in professional development for faculty or training for students in teaching roles. In contrast, SEC D2 functions primarily as a hub, aimed at communication within the institution to increase awareness of opportunities, while connecting and linking individuals to these. Therefore, SEC D2 does not function in areas of curricular development, expanding use of evidence-based instructional practices, or training of students for teaching roles (e.g., LAs, TAs). Cross-case findings for each area will be developed below, beginning with educational research, which emerged as a role of primary importance to each SEC.

### Educational research in undergraduate STEM

Engagement in educational research is one of the most important mechanisms through which these SECs contribute to undergraduate STEM education. Additional file 3 contains descriptive data for each institution across organizational level, and is used below to contextualize the importance of educational research for each center. All comments referenced in this section are from Additional file 3. Common areas of research include discipline-based educational research (DBER), scholarship of teaching and learning (SoTL), and individual action-research projects with faculty and departments. Specific research projects are focused in a variety of areas including the design of curricular interventions aimed at improved student engagement and success (e.g., course redesign, the study of peer-mentor interventions), generation of discipline-based educational theory aimed at improved learning outcomes within specific disciplinary contexts, and studying the implementation and outcomes of evidence-based instructional practices. In each of the six cases, SECs lead STEM education research efforts on their campuses through their many grant, philanthropic, and/or business/industry-funded initiatives, while also assisting faculty in submitting competitive proposals to continually renew and expand these efforts. “Here at (Center name) we have 16 externally funded projects underway” (Communications Director, Institution B1). Case analyses show that SECs catalyze departmental efforts by awarding small grants (seed funding), and by engaging faculty in center-funded research projects. The data show that at five of the six institutions, these efforts spawned larger departmental initiatives. For example at Institution B1, “The seed grant from (Center name) provided the spark that led to momentum and NSF funding” (STEM faculty). At Institution F3, the director was awarded a National Science Foundation grant for the purpose of increasing the up-take of evidence-based instructional practices across STEM departments. This funding was used to encourage departmental efforts. “The (NSF grant name) brought resources and helped us to engage faculty, and it slowly spread throughout our gateway courses” (Department Chair, Institution F3). This chair further described how the use of evidence-based instructional practices by his department and one other had begun to spread across disciplines in introductory courses, as a result of these initial efforts enabled by the external award acquired by their SEC. This theme is present throughout the case data: resources distributed by SECs allow small efforts to expand and engagement to spread (Additional file 1, row 1a).

Cross-case data show that the ability of SECs to earn funding enhanced their credibility, as well as that of STEM education research, on their campuses (Additional file 1, row 1b). This is described by a STEM department chair at Institution E3, “The Center has a pretty big profile here in our college. It is quite a big deal, because it is funded well and that’s the gold standard everybody uses. You convince someone that your work is fundable, that legitimizes it to a large degree. I don’t believe it’s just funded nationally; I believe the state of (state name) is also funding it.” Faculty comments show they find it advantageous to be associated with their SEC (Additional file 1, row 1c): “Being affiliated with (Center name) has stature associated with it and I benefit from being able to reference my work with (Center name)” (STEM faculty, Institution B1).

Each of the SECs use their educational research expertise to assist departments with the assessment and evaluation of discipline-based educational methods/innovations/reforms on undergraduate student learning and success (Additional file 1, row 1d). The Vice Provost at Institution E3 values the data provided by the SEC, because it informs important decisions regarding the success and scaling of intervention strategies. “(Center name) provides data to the university about undergraduate STEM, so that we can make informed decisions, and that’s a really key role of the Center.” In addition to assessing initiatives and curricular innovations, four SECs also partner with the office of institutional research to obtain and analyze data to inform STEM department and administrative decisions: “We rely on the data analytics provided by the Center, which assists us in focusing our efforts” (Provost, Institution F3). SECs engage in this partnership to understand STEM-specific need areas, helping them to allocate center resources effectively.

**Fig. 1:**
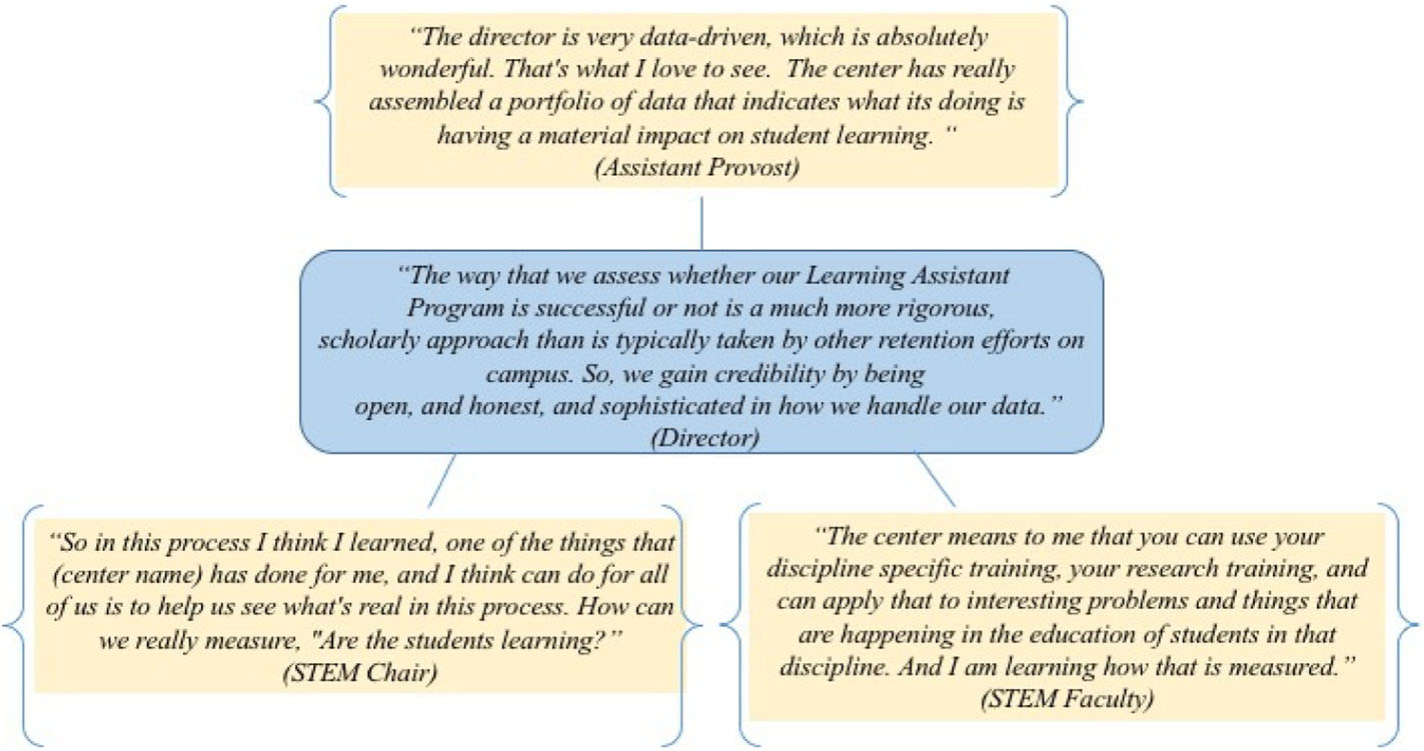
SEC role in educational research. A representative example from Institution E3

In the example shown in Fig. 1, the SEC director describes how the research carried out by the SEC is perceived to enhance the quality of assessment, and his description is supported across levels. The Assistant Provost values data to corroborate student success, and the department chairs and faculty appreciate gaining a deeper understanding of how to think about and measure student learning. Across the cases, research and associated assessments carried out by SECs were recognized by faculty and administrators as a key component of center expertise. Further, the descriptive nature of our data show that directors and staff apply their skills in ways that are complementary and sensitive to the disciplinary approach required for understanding teaching and learning within STEM departments. Through their partnerships with faculty, SECs provide advice and iterative feedback to guide the measurement of learning outcomes. Common themes noted by STEM faculty across institutions demonstrate that faculty rely on the SEC to assist them with data interpretation: “(Director’s name) has always been available as a person that we would discuss things with. ‘What are we doing here? What are we learning? What should we do next? How do we know if this is valuable?’ These are the kinds of conversations that everybody engaged in (NSF grant name) has had with (director’s name) over the years” (STEM Faculty, Institution F3). A department chair at Institution B1 notes the department’s reliance on the SEC to assist them in developing assessments, “What’s a meaningful measure of assessment when you’re doing something new from the ground up? That’s where we’ve engaged with (Center name). They advise us in this area.”

Cross case data show that upper administrators value SEC partnerships with faculty in areas of educational research, and recognize the importance of this blended expertise. “Yes. This is one of the problems sometimes… there’s faculty in the disciplinary departments who have an interest in this, but many of those folks don’t have the research skills in what is functionally social science, not math and physics. It’s a different way of doing research” (Dean of Natural Science, Institution B1). Faculty also acknowledge their need for educational research support. As one faculty member from Institution C1 explains, “Yeah, so it took a long time, then we started generating data and none of us have any educational data expertise and that is really when (director’s name) joined the group, because we needed someone from the science education side to sort through all the data.” At Institution E3, the Vice President for Research explains, “So the Center has brought a level of quality control to the larger institutional efforts around STEM education reform. If I had to make a recommendation it would be that more universities could benefit from having a (Center’s name) around. Because you have a lot of well-meaning faculty, and good, excellent lecturers, know the material, care about students, but aren’t necessarily education researchers. There is sort of a level of unintended quality control that gets built in.” This sentiment is echoed by upper administrators across cases, suggesting the SEC’s research expertise is perceived to keep the bar high for scholarship in this area (Additional file 1, row 1e).

Each of the SECs had a core community of affiliated faculty. This interdisciplinary community consisted of faculty from various STEM departments, some of which were engaged in DBER, others in current SEC projects (i.e., grant initiatives or seed-funded awards), as well as new faculty interested in learning about DBER research and/or curricular innovation. As faculty participated in various research projects and initiatives, the SEC became a meeting place for them to share ideas, thus promoting dialog around student learning (Additional file 1, row 1f). Across all SECs to varying degrees, our findings show this supported the growth of faculty identities in the scholarship of disciplinary pedagogy, which increased feelings of self-efficacy and job satisfaction (Additional file 1, row 1 g). “Through my involvement with the Center, I have found a group of like-minded colleagues, that I did not have within my own department.” (STEM Faculty, Institution B1), “The Center has validated my research interests and I feel that this research is important, just as important as my granular research in (STEM topic)” (STEM Faculty, Institution E3). This emerges as an important way that SECs impact the overall culture on their campuses.

In summary, the results show that SECs engage in research for the following reasons: (a) to bridge the research to practice gap and improve student learning, (b) to enhance pedagogical content knowledge, and (c) to design, implement, and assess curricular innovations. In parallel with these research efforts, SECs assess and evaluate to (a) measure the success of curricular innovations, (b) provide proof of efficacy and value, (c) support partnerships that require these services, and (d) earn funding. Across each institution studied, the data show that SECs’ prominent role in educational research is instrumental in encouraging the engagement of STEM departments and faculty in the improvement of teaching and learning (Additional file 1, row 1 h).

### Enhancing the quality of teaching and learning

In addition to SEC roles in educational research, Table 4 shows a variety of programs and services offered to both faculty and students that strengthen teaching and learning. Additional file 4 provides representative examples of the ways these SECs enhanced the quality of teaching and learning, across institutions and organizational levels. All comments in this section appear in Additional file 4. Each of these SECs broadly promotes and facilitates the use of evidence-based instructional practices (EBIPs) either directly as a result of their programs and services or indirectly through their research. The results show that SECs provide impetus for a greater investment in EBIP’s (Additional file 1, row 2a). This impetus primarily arises from increased resources, both human and financial, made available through the SEC. The primary impact on teaching and learning among and within disciplinary departments is through professional development arising from externally funded STEM initiatives, DBER research projects, and curricular reform efforts. In four of the six SECs studied, the professional development is directed toward the implementation of EBIPs to strengthen disciplinary curricula. In interviews, faculty describe an increased understanding of student learning, and the value of coming together to discuss teaching (Additional file 1, row 2b). As a STEM faculty member who is engaged in a learning community organized through their SEC describes, “I knew that I may never be at the level of say John or Ben where that is my full-time job. I have to run a research lab, I have to get grants, I have to do this stuff. But there was always an incentive to come, because it made my teaching easier. And I knew I could do things that would make it easier, meaning if they understand it more, you have less frustrated kids showing up in your office going, ‘I do not understand’” (Institution C1). Faculty also explain they “want to be a more effective teacher,” (Institution A1) and this was linked to a more enjoyable classroom experience. “I wanted to have more fun as a teacher. I wasn’t having that much fun anymore. I think I was doing the same thing all the time, just standing up there for an hour and lecturing and for me that was starting to get stale. I thought it was starting to get stale to the students as well” (STEM faculty, Institution B1). Cross-case data show SEC-led initiatives collectively engaged faculty in a number of ways that led to improvement in their quality of teaching (Additional file 1, row 2c). “I went from doing okay, I was never I’d say a bad teacher, to doing much better. In the end, we stayed with it because we think it does work. Not only for the students, but also for us” (STEM faculty, Institution C1).

The data also suggest that faculty are committed to improving their teaching when the SEC partners with them to provide guidance and expertise (Additional file 1, row 2d). As one associate director explains, “we needed something close to home staffed by people who would be interacting with our faculty on a day-to-day basis, that our faculty would understand from the very beginning was directly relevant to their everyday teaching” (Institution C1). This partnership frequently involves the measurement of learning outcomes to evidence the result of EBIP strategies. As the Director at Institution A1 describes, “and this leads into evaluation, because you can’t expect most faculty to try them (EBIPs) unless you help evaluate them right?” Data in five of the six cases show that departmental discussions of teaching led to curricular reform that sparked on-going discussions and questions, as departments began to consider the progression of concepts throughout the undergraduate curriculum (Additional file 1, row 2e). “So we as a group try to work together to try to say, the people teaching the upper level classes, like immunologists, ‘What are the three things you want students to know coming into your class?’ And so that kind of got transmitted back to people teaching introductory microbiology. We kind of tried to integrate the classes across, and then we used the concept inventory to look at how well students did, what were their misconceptions when they entered the class and then are we getting rid of some of these misconceptions and that was the cool thing” (STEM Faculty, Institution C1).

Additionally, four SECs continue to seed the use of EBIP’s through training programs for peer mentors (e.g., Learning Assistants), graduate teaching assistants, and post-doctoral associates, further increasing student potential for success and retention within STEM (Additional file 1, row 2f). The focused support offered by these SECs was initially tied to externally funded initiatives. As word of their effectiveness spread, these initiatives also received internal funding, and evolved to reach across departments. As the director from Institution A1 describes, “If we care about undergraduates, it means we need to offer professional development for graduate students. We have a course in the fall semester, which is mandatory for all graduate students. It’s two hours per week. This includes one-on-one observations of each other (teaching) and opportunities for faculty from their department to come and observe them. It has been very successful. A few department chairs have come to us to request it.” These SECs organize and staff peer mentor training, and also bring faculty together to share in the process. In this example, the SEC works with an affiliated STEM faculty member to staff the Learning Assistant program, “We typically have about 30 to 35 learning assistants every semester. The way that it’s set up is that LAs take a course in pedagogy during their first semester as learning assistants. The way the pedagogy course is structured, they get a lot of practice-based things, how to ask open-ended questions, how to engage with student groups, dialogic discourse, these kinds of things that are very much about classroom management and how to work with students. Then we focus on learning theory” (STEM faculty, Institution E3). The results show that faculty who utilized peer mentors felt the training that students received made the programs more impactful, for both the mentors and the students (Additional file 1, row 2g). In three cases, there was evidence to suggest that faculty who used peer mentors were more committed to the use of evidence-based practices (Additional file 1, row 2h).

Cross-case administrative comments, shown in Additional file 3, corroborate comments made by faculty and SEC staff in the areas mentioned above. These comments collectively reveal that SECs are valued for the way they promote the importance of teaching (Additional file 1, row 2i). As the Dean of the College of Science at Institution C1 explains, “There’s a real culture of respecting the teaching faculty. The Center promotes this.” Administrators describe the contributions made by their SECs as a key component in the implementation of evidence-based instructional practices. They also underscore the resources provided by their SECs to support this process. Importantly, administrators also recognize that these efforts require continued focus over time. “That was a long effort; over several years we moved into action research, and then education research. There are faculty learning communities. There was just a whole range of programs” (VPR, Institution F3). Administrators look to faculty associated with the SEC to lead transformational learning, as the Vice Provost of Academic Affairs at Institution E3 explains, “to take approaches to learning that have been successful, that a faculty member who’s been associated with (Center name) has experienced, and try to then expand it to other sections of the same course taught by other faculty.” Case data show that SECs offer support to facilitate the scaling of successful interventions (Additional file 1, row 2j).

Another way SECs contribute to the institutional culture for teaching and learning is through the leadership of affiliated faculty. These faculty are engaged with the SEC as research scholars or as partners in the use of EBIPs (Additional file 1, row 2k). They have the important role of modeling and advocating for the use of best practices within their home departments. In five of the cases, affiliated faculty lead and support departmental efforts to improve teaching by partnering with colleagues, while setting an example for the use of effective pedagogical practices. For example, at Institution F3, a team of faculty partnering with their SEC on an NSF grant were responsible for bringing EBIP strategies back to their respective departments. As the biology chair describes, “So biology’s been implementing this. We started last year and it sort of continued into this year. It works out to be about every other week we do something in our department meetings, and its sort of a combination of discussing EBIP strategies and people sharing examples of what they do in their classes” (Institution F3).

Across the various sites, SECs play an important role in disseminating the results of research studies carried out at their local campuses, as well as providing a scholarly base for DBER and more broadly the scholarship of teaching and learning as it applies to the STEM disciplines (Additional file 1, row 2l). In four cases, SECs were relied on for high-quality research to support student learning. “We rely on the Center and the people who participate in (Center’s name) to make sure we have a strong scholarly base for what’s presented in the principals of teaching workshops. To me, the crucial element there is to pay attention to learning” (Dean of Undergraduate Education, STEM faculty, Institution B1). Local dissemination of studies often served to spark interdisciplinary conversations, which informed reform efforts in gateway courses. Different modes and methods of dissemination were important to faculty engagement. Institutes several days in length, engaging faculty longer than a workshop or seminar, were effective for some. “To sit there for two and a half days and have them put up - this is what the research is showing us and it’s black and white, what the research is showing us… and I don’t think it would work in an hour. I had to be immersed in it from different dimensions and see that there’s many different techniques. Everything that they present is based in research-based findings” (STEM faculty, Institution A1). Interview comments collectively show a wide variety of faculty participation in these seminars/institutes/workshops, ranging from senior to junior, tenured, as well as teaching track (Additional file 1, row 2m).

### Broadening participation and institutional capacity for STEM learning

Each SEC worked to broaden participation and institutional capacity through a variety of different functions, shown in Table 5. This table displays over-arching similarities between SEC functions, as well as illustrating contrasts, in these areas. For the most part, contrasts in SEC functions can be attributed to the degree in which they were tailored to: meet the needs of diverse student groups, provide an infrastructure for broader impacts, and support the K-16 pipeline. SECs D2, E3, and F3 were most similar in their support for students, and this carried over into their role in broader impacts (BI). SECs A1 and C1 were the least engaged in each of the functions that support the K-16 pipeline, while SECs D2 and E3 were the most engaged. Cross-case findings for each of these areas will be developed below, beginning with building partnerships for research and community, a core role in which all SECs were engaged. Additional file 5 provides full representative examples of the ways these SECs broaden participation and institutional capacity across institutions and organizational levels. Descriptive data from Additional file 5 was used to contextualize the importance of this role across SECs.

**Table 5:** SEC functions directed toward broadening participation and institutional capacity in undergraduate STEM

Function	Institution/Center					
	A1	B1	C1	D2	E3	F3
Build partnerships and community for STEM Ed learning and research	x	x	x	x	x	x
Establish relevance for STEM learning and meet student needs:						
• Connect students to opportunities (e.g., URE’s)				x	x	x
• Host events to increase awareness and interest	x	x		x	x	x
• Provide authentic experiences		x		x	x	x
• Mentor training programs for faculty engaged in URE’s	x		x	x		x
• Diversity and inclusion training: workshops, seminars	x		x			x
• Provide community for students				x	x	x
Infrastructure for broader impacts						
• Provide a home				x	x	x
• Coordinate				x	x	x
• Position competitively for funding		x			x	x
Support for K-16 pipeline						
• Improving K-12 instruction	x	x		x	x	
• BI and outreach activities		x		x	x	x
• Facilitate 2- to 4-year program transitions			x	x	x	x
• Bridge programs				x	x	x

x = evidence triangulated within and across administrative levels for that function

### Build partnerships and community for STEM Ed learning and research

Cross-case data analysis suggests that the blend of STEM expertise, with a centralized “hub” for STEM activities, and a network of partners contributed to SECs’ unique ability to expand institutional capacity. Building partnerships and community consisted of (1) connecting faculty with similar and complementary interests, (2) connecting faculty to available resources, as well as (3) connecting upper administrators to faculty efforts. Each of these connections increased shared understanding, and often fostered engagement with the SEC allowing it to grow and strengthen. As a faculty member at Institution A1 explains, “There’s been a couple of other initiatives like that where the (Center name) is the nucleating organization reaching out to interested faculty and different groups to further conversations.” At Institution F3, a faculty member describes a new grant that brought in needed resources, “The Center director is the co-PI, she was a big part of bringing that grant in. We’re collaborating with a group of about 11 faculty right now with an extension to a larger circle of about 25 faculty collaborators.” SEC-hosted events bring together faculty, students, and upper administrators; in this way, they increase awareness of grass-root efforts within departments (Additional file 1, row 3a). “I know that almost everybody appreciates what (Center name) is doing now. The respect is there, faculty and students see that at the end of every year we have a poster session and we celebrate (Center name), and the Provost comes and talks at that. I go and welcome students. And so they see that it’s been recognized by the institution, and there is respect. It is nice because it keeps us connected to what the faculty and students are doing” (Dean, College of Science, Institution E3). Across the cases, the data show SECs showcase events that increase engagement and understanding, both of which are necessary to unify institutional efforts.

An attribute common to these cases is that each SEC director holds a PhD in a STEM discipline and is jointly appointed as a faculty member, facilitating their ability to build partnerships within STEM departments. The data show these SEC directors seek to bridge the different disciplinary priorities by bringing faculty together to share common goals and envision opportunities for synergies (Additional file 1, row 3b). Collaborations and partnerships developed through the SEC were key to establishing a viable network. Cross-case data shows SEC networks to be an important resource through which they contributed to the expansion of institutional efforts (Additional file 1, row 3c). “The way I think about it – it’s like building a map of where efforts are going on, and then I/we build a Network to link those efforts. I also ask ‘how can I make it better?’, and what can we share with the rest of campus” (SEC Director, Institution E3)? This important role is echoed by the VPR from Institution F3, who describes his institution’s need for this service. “This may not sound like a big deal, but it’s a big deal to me and it’s a big deal to the University that (SEC Director’s name) has the pulse of what’s going on. She is networked with what’s going on in the campus. She has a network, and the connectivity. I rely on the Center for that service, and I know others do as well.” Analyses show that these SECs are well positioned to build partnerships because they work across the disciplines developing broad awareness of current departmental efforts, interests, and strengths, which they interface with institutional needs. A description of how these SECs use their partnerships and community is described in the following sections, beginning with meeting the needs of students.

### Establishing relevance and meeting student needs

A number of SEC programs and services are directed toward supporting students, with specific attention to underrepresented students in STEM disciplines. This was true across all the cases studied and took shape in a variety of ways, as listed in Table 5. For example, SEC staff provide counseling and advice for individual and groups of students. “And, I would say probably that the main support for underrepresented students looks like connecting them to apply for learning opportunities. So, whether that’s undergraduate research positions, internships, professional opportunities, those kinds of things, to help clarify kind of what they want to do with their careers in STEM and help them engage at the university” (Center staff, Institution F3). See Additional file 5 for full comment. Five SECs hosted events to increase student awareness and interest in STEM and related fields (Additional file 1, row 3d). Examples of these include undergraduate research conferences, local industry and business symposia, sometimes linked to broader impact activities, and organized peer to peer community building, all of which serve to promote interest and retention in STEM. Our case data show extensive engagement of three SECs in areas that provide authentic experiences for students (e.g., business/industry partnerships and undergraduate research) (Additional file 1, row 3e). In addition, these SECs frequently support students by offering funding for these opportunities through externally funded resources (e.g., Louis Stokes Alliances for Minority participation, LSAMP).

Five of the six SECs offer mentor training programs for faculty involved with undergraduate research experiences (UREs) and internships. “Many of our programs require faculty to have training prior to becoming an undergraduate mentor. HHMI for example. We provide that, and we help match students to mentors” (Director, Institution D2). Additional representative examples are shown in Additional file 5. Along with effective mentoring strategies, these programs feature methods that foster faculty awareness of and sensitivity to the diverse needs of underrepresented students assisting them in providing a more inclusive experience for students from diverse backgrounds. As the associate director at Institution A1 describes, “I think too, that the number of workshops that we do, that are directly and consciously on inclusive teaching topics, like stereotype threat, growth mindset, and understanding bias. During a STEM Institute faculty are going to get these things mixed in.” Across these five cases, directors describe opportunities for new mentors to be trained in strategies that foster underrepresented student success (Additional file 1, row 3f).

Further, SECs D2, E3, and F3 provide community for students by hosting events and activities that bring students together to share about their undergraduate research experiences (UREs). These SECs gain valuable insight into the success of these formative URE experiences through their engagement with students. The data in these three cases show that SECs utilize their student networks to promote the benefits of these experiences to new students, who otherwise would likely have no exposure to this kind of opportunity (Additional file 1, row 3g). In this way, SECs play an important role in equitably linking students who are not aware, or empowered to advocate for such opportunities. “We bring students together to hear from one another. They see students like themselves explaining the value of these experiences” (Staff, Institution D2). See Additional file 5 for additional representative examples. Faculty comments explain that SEC-organized activities and programs for students make it feasible for them to take on more URE’s, because they can rely on the SEC to provide enrichment experiences for these students. “These experiences take time, and part of our responsibility is to provide community for participating students. I can now rely on the Center for this” (STEM faculty, Institution F3). Two institutions had numerous UREs, which their SEC helped to organize.

Additionally, to better meet student needs, SECs C1 and E3 design and administer exit surveys to gather data on student experiences in URE’s, which included questions on workforce preparedness. The results of these surveys are used to engage disciplinary departments in a discussion of their goals relative to students’ experiences. Initiating these discussions assists departments in making improvements, while encouraging more deliberate communication with students to achieve the desired outcomes. At Institution E3, SEC-affiliated faculty were focused on curricular reform designed to include career preparation. As one faculty member describes it, “There is so much natural synergy between how we talk about career preparation in a kind of comprehensive and expansive way. We want to integrate that into our efforts toward course transformation, so that students can see these connections” (Institution E3). See Additional file 5 for full comment. The data show that SECs support efforts directed toward improved student engagement through applied curricular experiences (Additional file 1, row 3h).

### Infrastructure for broader impacts

Our data show that three of the six SECs establish and maintain an infrastructure for broader impacts (Table 5), providing a home, as well as resources, for previously funded successful STEM programs and initiatives, thereby contributing to their continuation. “It’s so difficult, so many of these grant funded initiatives. You know, everybody follows the money to begin with, and then you’re left with how do you keep this good thing going? The Center provides a structure that helps us with this” (Sr. Assoc. Provost, Institution E3), and at Institution F3, “We are seeking to institutionalize the annual undergraduate research conference that we have held for the last ten years, and the Center is going to provide a home for this. Now this event will receive the recognition it deserves” (VPR, Institution F3). Within this infrastructure, SECs organize and host undergraduate research conferences, training for peer-led team learning assistants, outreach to K-12 and local communities, as well as community building experiences for undergraduates engaged in STEM programs at the home institution. This infrastructure benefits students, faculty, and the institution due to the cyclic fashion in which it expands existing efforts, providing new opportunities. The data show that faculty reference these activities and programs, as evidence of existing campus efforts, allowing them to submit more competitive proposals for external funding (Additional file 1, row 3i). In addition, this infrastructure makes it feasible for faculty to pursue larger grants. For those institutions whose SECs offer this type of infrastructure, it serves to expand institutional capacity in undergraduate STEM. As faculty bring in more funding, contributions to broader impacts grow, which increases support and opportunities for students (Additional file 1, row 3j).

### Support for K-16 pipeline

Case findings show that each of these SECs carry out function(s) to support the STEM pipeline (listed in Table 5). Roles in this area range from improving STEM instruction through professional development and curricular materials for K-12 to outreach activities that increase youth and public engagement. The Dean of Engineering comments on her partnership with the SEC at Institution F3, “So, last year we started an all girls first robotics team. I knew (Center staff member) was interested; I spoke with her and she took charge of it. But we hosted it in the College of Engineering and a couple of my faculty are mentors.” Several faculty describe engagement with their SEC to integrate innovative curriculum into the local high schools (Additional file 1, row 3k). “We are working with (Center name) to introduce this class into high school. We’re intending on going for a joint grant too, it’s a (grant name), because one of the main parts of the practice is computation, so we do a lot of computation in the class. Since that has become important in high school as well, we’re looking to get it funded to bring the course to (state name) high schools” (STEM Faculty, Institution B1). For Institutions B1, D2, E3, and F3, this was an important role, to increase the visibility of their STEM programs. “We have a large population of commuter students who have jobs. Most of which are first generation… about 85% of our students actually, so we put significant effort into K-12 and local community outreach. The Center plays an important role here. It helps to coordinate these opportunities and importantly it facilitates communication between schools and the university” (VPR, Institution D2). Additional file 5 has further supporting comments by administrators to show that these institutions are focused on expanding their student base through SEC outreach.

Additionally, SECs play an important role by working with students and faculty to facilitate transitions from community colleges. At SECs D2 and F3, this took place through direct mentoring associated with LSAMP funding and institutional initiatives, “I also, through LSAMP, work with community college students as they transfer, specifically at the College of Western xxx” (Director, Institution F3). At SEC C1, support for 2- to 4-year transfer students was tied to curricular reform associated with a grant awarded to the SEC. “The idea was to make their introductory microbiology course more comparable to ours, using the same case studies, to bring the same curriculum back and forth so that when students did transfer in, they would be able to come in more seamlessly” (STEM faculty, Institution C1). In this example, faculty had concerns about a particular group of community college students who had not been as successful as others. The SEC supported the department in gathering data to confirm this was indeed the case. Faculty then took steps to support this group of students and the SEC assisted in the development of necessary curricular resources. Refer to Additional file 5.

### Broadening participation through teaching

**Fig. 2:**
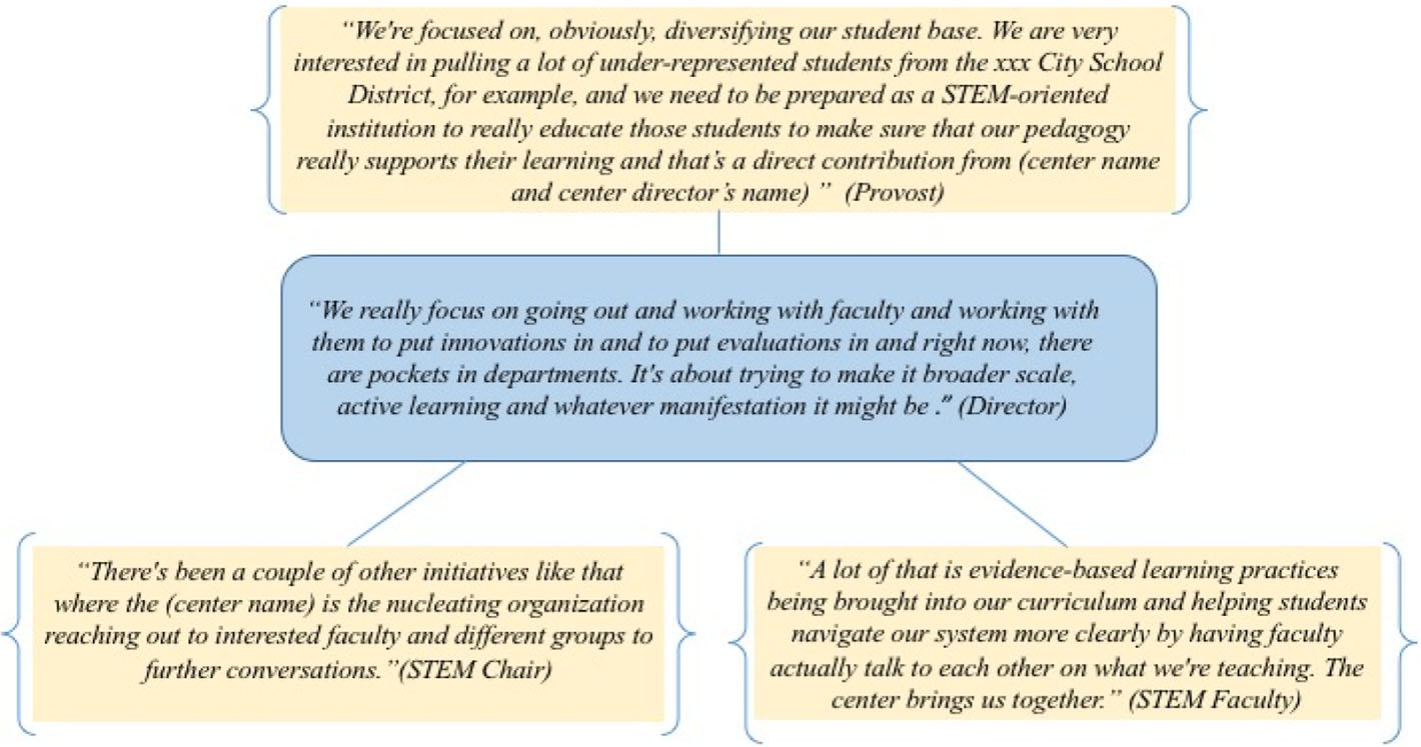
Improved teaching supports broader participation. A representative example from Institution C1

Administrative comments corroborate the value of SEC functions that contribute to institutional goals for broadening student participation in STEM. As these institutions seek to increase the success and engagement of a broad student demographic, SECs make important contributions. For example, as the Provost at Institution A1 explains, “what we are not doing is giving everybody the same fair chance to be a scientist. Part of the reason is because the methods that we use to teach - writing on the chalkboard and all that stuff is a 100 years old if not more. The Center is supporting our mission to improve in this area.” Case data provides evidence to suggest upper administrators associate teaching methods with equitable student learning opportunities. Likewise, comments like those shown in Fig. 2, highlight the ways in which SEC support improves teaching by building faculty partnerships that increase the uptake of evidence-based instructional practices. SECs play an integrative role by bringing faculty together and fostering discussion among a community of practitioners. Cross-case data show center functions that support improved teaching and learning are also perceived by faculty to broaden participation (Additional file 1, row 3l).

### Addressing national priorities

By framing our results in the areas related to (1) the improvement of STEM learning, and (2) broadening participation and institutional capacity for STEM learning, we identified center functions that pertain to our third research question. *In what ways are SECs addressing the national priorities in STEM education*? Detailed functions previously shown in Tables 4 and 5 allow us to describe the ways in which these SECs are assisting institutions in meeting some of the national priorities. A summary of our findings are presented in Table 6. The activities of SECs were organized into three areas described by the NSTC Federal 5-year STEM education plan (2013). Each of these are well aligned with the goals of institutional change initiatives described by the AAU Framework for systemic change in undergraduate STEM (2013), and the AAC&U Achieving Systemic Change Sourcebook (2014). Our research findings show that these SECs collectively contribute to our national priorities in STEM education by influencing the quality of undergraduate STEM education at their institutions. These SECs also assist departments, faculty, and administrators in understanding how their work is situated within the broader national framework to improve undergraduate STEM education.

**Table 6:** SEC functions contributing to national priority areas in undergraduate STEM

National priorities		
Improve STEM learning	Improve institutional capacity for STEM learning	Broaden participation and access
Provide faculty development support tailored to department and individual faculty needs	Collect and share data on STEM programs, initiatives, course innovations	Improve K-12 STEM instruction; Improve preparation for higher education
Carry out research studies to gather data and provide evidence at local institution	Seek and acquire funding to engage faculty and departments	Facilitate transfer from 2- to 4-year programs; Coordinate/manage bridge programs
Identify, develop, and implement effective EBIPs	Affiliated faculty engage in research and model best practices in home departments	Provide authentic learning experiences through URE’s, and local business/industry partnerships
Educate through seminars, institutes, and speaker series	Provide community for faculty engaged in educational research: DBER, SoTL, Action	Coordinate/carry out broader impact and outreach activities to increase youth and public engagement
Identify and support department exemplars; provide leadership	Sustain initiatives by providing infrastructure to support 1) Effective programs 2) Broader impacts 3) Established network	Provide diversity and inclusion programming

## Discussion

This study shares what was learned through the cross-case analysis of six STEM education centers (SECs). Through qualitative data analysis, we describe the primary areas these centers function in, and the contributions they make to undergraduate STEM education at their institutions. These areas are described across administrative levels to gain understanding of how center functions fit into the wider systemic landscape at their institution. Our research questions were best answered through a systems approach (Wasserman 2010; iTest 2013; Elrod and Kezar 2016; Miller et al. 2017) allowing us to situate center functions in context, through the perspectives of STEM departments and upper administrators, thus providing depth to our understanding of the research, programmatic, and organizational challenges in which these SECs were engaged.

**Fig. 3:**
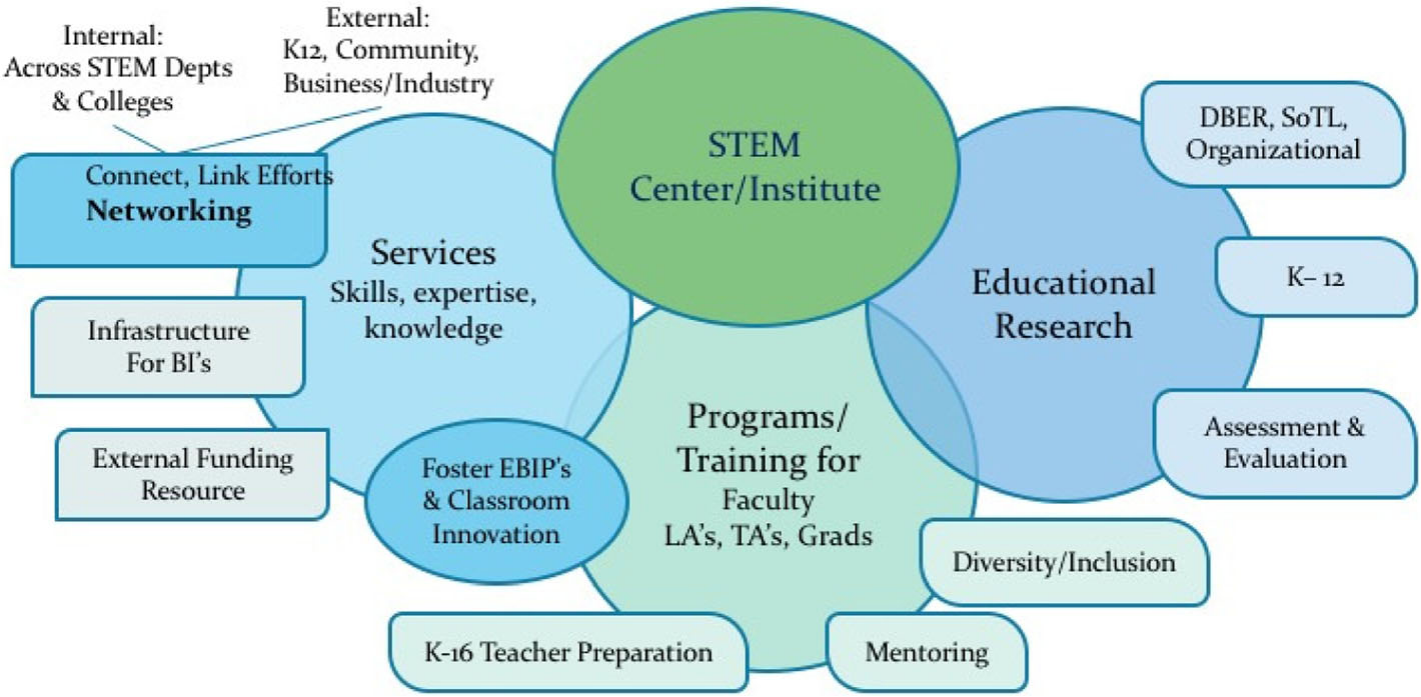
A model of STEM education center functions/roles

In this paper, we frame the work of SECs against the backdrop of national recommendations^2^ because the underlying need for SECs is situated within the context of an on-going national call for the improvement of undergraduate STEM education. Our findings describe the ways SECs are supporting their institutions by assisting them in creating an environment through which to encourage growth and improvement. SEC functions inform and unify current departmental efforts, while elevating their visibility and importance both internally and externally. Figure 3 summarizes SEC functions, grouping them broadly into three categories: educational research, programs, and services. Through their scholarship, SECs contribute to the knowledge base and provide funding, which adds resources and incentives for the implementation of EBIPs and educational research. SECs augment these efforts with the assessment and evaluation of learning outcomes and curricular innovations. Additionally, SECs act as an internal resource for faculty and instructors providing programs and training to foster the application of evidence-based instructional practices (EBIPs). Through their services, SECs play an important networking role, in some cases SECs provide the infrastructure for broader impact activities, and act as an external funding resource. Four SECs combined these roles to competitively position the institution for external awards. Our results show that SECs make key contributions to their institutional environments, and these play an important role in shaping intended outcomes, both prescribed and emergent, institutions have for undergraduate STEM education reform (Henderson et al. 2010).

### Setting-up the environment

Fundamentally, these structures centralize STEM activities and initiatives, which facilitates communication and assists in building partnerships. Overall findings show that SECs contribute to the status and importance of knowledge related to the educational aspects of STEM teaching and learning. Acting as a centralized entity, while carrying out their own research, programmatic, and organizational functions, SECs exhibit the ability to integrate their research in ways that stimulate the implementation of teaching and learning initiatives. When seeking to understand the roles of SECs, it is important to consider the fact that STEM disciplines are notoriously perceived and self-described as unique and specialized entities that require disciplinary expertise to navigate (DBER report 2012). Our data show that an integral part of what these SECs do is to work within and across disciplinary boundaries, and therefore implies they possess the necessary expertise. “You can’t be a generalist and really do the kinds of things we’re aiming to do. It gets you so far, but it doesn’t get you everywhere you want to go” (Director, Institution C1). Our findings show many ways in which the functions of these SECs exemplify recommendations from the DBER report (Singer et al. 2012), summarized in Table 7.

**Table 7:** SEC engagement in areas aligned with DBER report

• Provide community for DBER scholars, and for all faculty engaging in EBIPs	
• Provide resources and incentives through external awards	
• Engage STEM faculty in the use of EBIPs	
• Partner with STEM departments/faculty to measure the success of curricular innovations	
• Scholars affiliated with SECs model best practices in home departments	
• Disseminate the results of STEM education research through seminars, institutes, research collaboratives, and learning communities	

### Discipline-based educational research and SECs

Each of the six SECs is actively engaged in the areas shown in Table 7. First, they provide community for DBER scholars and faculty interested in the scholarship of teaching. This community serves to maintain a core group of interdisciplinary faculty who engage in on-going “sense-making” activities, shown to be critical to the cultural change process, giving grass-roots initiatives a foot-hold within departments (Kezar 2013). While our data do not reflect the degree of participation within each of the STEM departments, there is ample evidence to show that SECs regularly bring together cross-disciplinary STEM faculty to share and support one another’s work. Faculty comments describe the ways in which SECs validate their research interests, thereby increasing their job satisfaction and self-efficacy. Second, SECs bring in resources through externally funded awards, which provide credibility for DBER scholars. In addition to increasing credibility, external funding resources provide incentive, helping to catalyze teaching and learning initiatives within departments and increase opportunities serving to broaden the participation of students. Third, SECs play an influential role in the adoption of enhanced teaching practices, by engaging STEM faculty in externally funded projects that necessitate the use of EBIPs. In addition, some SECs offer small grants to faculty and departments to integrate student-centered practices, and engage in curricular reform. Our findings show that these small grants catalyze efforts that continue to expand, some leading to successful grant proposals that further sustain these efforts. As noted by a STEM faculty member, “The seed grant from (SEC’s name) provided the spark that led to momentum and NSF funding” (Institution B1). Fourth, faculty, departments, and administrators rely upon SECs for high-quality assessment and evaluation of curricular innovations and learning outcomes. Studies have reported the lack of assessment support as a barrier to shifting teaching norms in STEM departments to include more “active learning” strategies (Shadle et al. 2017). Our research suggests SECs’ willingness to partner with faculty to assess discipline-specific learning outcomes influences their engagement and interest in adopting EBIPs. Fifth, DBER faculty associated with these SECs often serve as models for best practices in their home departments. These DBER faculty lead by example, acting as resources for others seeking to learn about the application of EBIPs, and the measurement of student learning outcomes. Importantly, they contribute to the departmental culture by placing an emphasis on teaching and learning, bringing contrast to the heavy focus on granular research. Finally, SECs also disseminate the results of DBER research, as well as other forms of STEM education research, across STEM departments through seminars, institutes, research collaboratives, workshops, and learning communities. These functions serve to build faculty awareness and interest, while connecting them with others with whom they can pursue and explore their ideas.

### Fast lane approach

As a result of SEC contributions to the institutional environment, a “fast-lane” approach toward institutional goals in undergraduate STEM may be realized. Our findings collectively suggest that SECs serve to catalyze teaching and learning initiatives that enhance undergraduate opportunities in STEM. This was evidenced broadly by themes within and across levels revealing that center functions collectively serve to stimulate activity. For example, dissemination events hosted by SECs increase faculty and department interest in educational research and curricular reform. This interest is further supported by small seed grants, partnerships for assessment, and faculty development for the use of EBIPs. Further, as these efforts grow, SECs also offer support and advice for the submission of grant proposals, and in some cases infrastructure for broader impacts. These examples show a clear progression of the ways that SEC functions can contribute to the initiation of new endeavors, and add momentum to current efforts. Across the six institutions, our data show that as engagement grows, a larger number of faculty had their accomplishments actually recognized, which often leads to further buy-in.

### Challenges faced by SECs

In studying these SECs, we identified some areas of challenge that may influence their ability to have a sustained impact on their institutional environments. These include reliance on external funding; specialized fields of science, technology, engineering, and math, collectively grouped homogenously as “STEM”; and competition with other centers and STEM departments for institutional funding. Our study did not explore these in any depth. However, they provide insight for institutions seeking to add or strengthen an existing center structure, and suggest possible directions for future research. First, institutions rely on their SECs to provide resources through externally funded awards rather than directly allocating institutional resources to these priority areas. Institutions appear to be slow to commit to the institutionalization of programs and initiatives shown to be successful. This hampers the ability of SECs to provide staffing to expand successful programs. Three of the SECs studied share concerns for retaining qualified staff due to their reliance on external funding. Second, STEM is a collective term used to encompass very specialized disciplines of science, technology, engineering, and math. To work closely with faculty and departments within each of the disciplines, SECs require staff with diverse STEM backgrounds, and/or affiliated faculty from a wide variety of disciplines, to broaden their support to each of those fields. Our cases show that SECs had engagement in STEM departments that were either aligned, or closely aligned, with their director’s area of expertise. Each of the SECs was actively focused on expanding their reach across the many diverse STEM disciplines. This presented a challenge, because SECs had a limited number of staff, therefore all STEM fields were not equally engaged and able to benefit from center resources. Some SECs address this challenge by establishing partnerships with a wide variety of disciplinary faculty. However, these SECs also reported difficulties in gaining departmental release time for these faculty. Additionally, at four of the six institutions, organizational structures placed SECs in direct competition with STEM departments, and/or other Centers (e.g., a center for diversity in engineering) for institutional funding; this impacts potential collaborations, making it difficult to unify efforts. This is by no means an exhaustive list of the challenges faced by SECs, but rather those that we identified as common to three or more cases during our analysis.

### SECs and institutional change

As noted previously in the background section, the Keck/Project Kaleidoscope Guide to Systemic Institutional Change in STEM Education (2016) was used to inform our inquiry into the roles of these SECs. This guide emphasizes the need for research, which extends “our knowledge of interventions beyond the department level” to support and develop institutional visions for undergraduate STEM education. Our findings suggest that the contributions made by SECs toward the research, programmatic, and organizational challenges facing undergraduate STEM education are such interventions. Through the systems approach taken here, we gain perspective on center functions, thus informing the way in which these structures can be utilized by other institutions seeking to achieve their own STEM education goals. For institutions seeking to use the Keck/PKal guide, our results show that SECs would be important partners to include at each stage of the *Model for Systemic Institutional Change in STEM Education* (Elrod and Kezar 2016, p. 10), because their functions have significant overlap with the suggested processes. For example, when conducting a landscape analysis to guide institutional decisions, SECs such as those at institutions A1, B1, C1, E3, and F3 have explored baseline data regarding student success in STEM courses and pathways, and each of the six SECs has knowledge of capacity based on their partnerships with faculty and departments, as shown in Table 4 of the results. This knowledge includes where current efforts are located, who is engaged, and the nature of these efforts; thus SECs can contribute valuable insights to inform institutional goals in undergraduate STEM. In five of the six SECs studied, we found “bottom-up” support from faculty, “mid-level” administrative support from department chairs, and “top-down” administrative support for their Center’s role on campus. This multi-level support has been identified as a “key factor for success” during the institutional change process (Abell and Lederman 2007; AAC&U 2014; Elrod and Kezar 2016; Miller et al. 2017). One of the strengths of our research design was that we were able to observe the ways in which these various levels of support reinforced one another.

Our findings illuminate the ways in which faculty and departments engage in SEC functions, as well as the perceived value of SECs by the upper administration. This triangulated view allows us to offer some observations about how SECs help their institutions to learn, change, and adapt to improve undergraduate STEM education*.* Through their roles in educational research and assessment and evaluation, SECs provide data to assist STEM faculty, departments, and institutions in learning about the ways in which current teaching and learning innovations support student success. For example, they provide data to inform decisions about which interventions to scale. SECs also assist with implementation by providing both human and financial resources that incentivize reform. Our findings suggest that over time, SEC contributions will lead to adaptations that place an increased emphasis on teaching and learning, allowing institutions to better meet the needs of their students in undergraduate STEM.

A critical component of what SECs do is to support institutional goals through their understanding of teaching and learning efforts within and across STEM departments, and at other institutions nationally and internationally*.* The very nature of their work necessitates SECs acquire a well-informed understanding of individual faculty interests and departmental goals that may serve as leverage points within STEM departments. Our findings show SECs frequently identify these through their roles in educational research, and evaluation and assessment. Additionally, as SECs seek to unify initiatives they gain awareness of STEM department interests, as well as areas of need. Identifying these potential points of entry helps the SEC to consider ways to support and grow STEM education initiatives, which can be linked to funding opportunities, and thus serve to position the institution more competitively. Through their disciplinary partnerships, SECs bridge the siloed existence of departments, and foster the integration of teaching and learning efforts. Our findings support a recent study published by the AAU STEM Initiative (Miller et al. 2017), which acknowledges the importance of “support structures,” that effectively align with department-based instructional improvement efforts. Here, we provide specific examples of the ways in which SECs act as support structures at the institutional and departmental level, the benefits of which are recognized by both the administration and faculty.

### Limitations

Because there are so many different types of SECs, our purposive sample is not representative of all SEC types. Nonetheless, this study offers specific insights as to the role of these SECs on their campuses, emphasizing functions common across sites. Our purposive sample was comprised of institutions that were looking to take advantage of a perceived niche area, STEM education. Each institution identified STEM education as an area of primary importance to their mission. Therefore, a STEM education center was aligned with their identity. Our sample did not include SECs with a strong focus in K-12 outreach or K-12 teacher preparation and thus does not capture features of these SECs and their significant contributions to STEM education, at their postsecondary institution.

## Conclusion

Our research provides insights across organizational levels, through which to understand how SECs function on their campuses. This deeper understanding is valuable to the network of STEM education centers (NSEC) organizers as they seek to facilitate the ways in which SECs learn from one another, provide collective leadership, and influence policies in STEM Education at the national level. This study also informs institutions seeking to improve undergraduate STEM education through the use of a center or institute. For example, it may provide information to organizational leaders as they seek to effectively utilize leverage points to influence the role of SECs on their campuses.

As established earlier, STEM education centers are symbolic of institutional commitment. Scholars as well as leaders in higher education recognize the benefits of such structures. In *The Accelerating Pace of Change in Higher Education*, Richard DeMillo explains “Systemic problems often demand structural solutions” (DeMillo 2017), and our findings suggest that SECs are structures that facilitate organizational processes in ways that accelerate the pace of reform in STEM education.

Our results show that the centralization of STEM education efforts through an entity such as an SEC allows for the integration of disciplinary research with curricular initiatives and related programs and services, to support departmental and institutional goals. As universities and colleges struggle to meet the national priority areas in STEM Education (NSTC 2013; AAC&U 2014), our findings show that STEM education centers are making meaningful contributions, and that it is not only the areas they are working in but also the way in which they integrate these that makes them powerful support structures for undergraduate STEM education on their campuses.

### Suggested areas for future research

Research which improves our understanding of the different phases of center evolution, from inception to maturation, would help to support the development and sustainability of SECs. Many SECs begin with momentum provided from external funding and as such benefit from an initial novelty period. To better support SECs, further information on the challenges noted previously: (1) reliance on external funding; (2) specialized fields of science, technology, engineering, and math, collectively grouped homogenously as “STEM”; and (3) competition with other centers and STEM departments for institutional funding, and their impact on SECs’ would be of value.

Our future research includes a national survey of SECs and Centers for Teaching and Learning to extend the generalizability of our site visit data, further establishing areas of impact and areas of need, such that these centers may be best supported through NSEC, as well as by one another, funders, and policy makers.

## Additional files

Additional file 1: Findings and occurrence in data source. (DOCX 80 kb)

Additional file 2: Center mission and upper administrator perspective. (DOCX 73 kb)

Additional file 3: Cross-institutional examples of the ways in which centers engage in educational research. (DOCX 74 kb)

Additional file 4: Cross-institutional examples of the ways centers enhance the quality of teaching and learning. (DOCX 73 kb)

Additional file 5: Cross-institutional examples of center functions which broaden participation and institutional capacity in STEM learning. (DOCX 74 kb)

## Abbreviations

BI: Broader impacts
DBER: Discipline-based educational research
EBIPs: Evidence-based instructional practices
NSEC: Network of STEM education centers
PCK: Pedagogical content knowledge
REU: Research experience for undergraduates
SECs: STEM education centers
SoTL: The scholarship of teaching and learning
STEM: Science technology engineering and math
UREs: Undergraduate research experience
VPR: Vice President for Research

## Footnotes

1 Carnegie classification: R1 (highest research activity), R2 (higher research activity), R3 (moderate research activity)

2 National priorities were based on those described by AAC&U CRUSE (2014), AAU Undergraduate STEM Education Initiative (2013), NSTC Federal 5 Year STEM Education Plan 2013.
