# Supplementary material for: STEM education centers: catalyzing the improvement of undergraduate STEM education
Source: Int J STEM Educ. 2018 Nov 12;5(1):47. doi: 10.1186/s40594-018-0143-2 (PMC6310466; doi:10.1186/s40594-018-0143-2)
Supplement: Supplementary file 1 — Findings and occurrence in data source. (DOCX 80 kb) [file 40594_2018_143_MOESM1_ESM.docx]

Additional file 1. Findings and occurrence in data source

Center = STEM education center (SEC)

Across three levels indicates presence in (1) center, (2) administration, (3) faculty/departments

Occurrence in data, indicates presence of finding. Presence in any level (e.g., faculty) indicates that it was evidenced by a minimum of three individuals at a given institution during interviews

Key:

Green = highlight indicates across all three levels at an institution

Blue = highlight indicates across two levels at an institution

White = indicates across one level at an institution

|  | Finding:  Case data | Data source occurrence:  Institution/Center |
| --- | --- | --- |
|  | ***Section 1: Educational Research*** | **p. 11** |
| 1a | The data show **resources distributed by the Center** allow small efforts to expand and engagement to spread. | A1, B1, C1, E3, F3  D2 / faculty and Center |
| 1b | The **ability of Centers to earn funding enhanced their credibilit**y, as well as that of STEM education research broadly on the local campuses. | B1, C1, E3, F3  A1/ Center and administration  D2/ Center |
| 1c | Faculty comments show they find it **advantageous to be associated with the Center** | A1, B1, C1, E3, F3/faculty only  D2 absent |
| 1d | Each of the **Centers used their educational research expertise to assist departments** in assessing and evaluating discipline-based educational methods/ innovations on undergraduate student learning and success.  e.g. research and associated assessments carried out by the Center were recognized by faculty and administrators, as a key component of Center expertise.  e.g. faculty rely on the Center to assist them with data interpretation | A1, B1, C1, E3, F3  D2 absent |
| 1e | The Center’s research expertise is **perceived to keep** **the bar high** for STEM education scholarship | A1, B1, E3, F3  C1/ faculty and Center  D2 absent |
| 1f | The Center was **a place** for faculty **to come together** and share ideas, **promoting dialogue** around student learning – community – growing organically | A1, B1/ Center and faculty  C1, D2, E3, F3 |
| 1g | The Center supported the growth of faculty identity in the scholarship of disciplinary pedagogy, which **increased feelings of self-efficacy and job satisfaction**. | A1/ faculty  B1, D2, E3  C1, F3/ faculty and Center |
| 1h | The data show that Centers prominent role in **educational research was instrumental** in encouraging the engagement of STEM departments and departmental faculty in the improvement of teaching and learning | A1 – F3 (all Centers) |
|  | ***Section 2: Enhancing the Quality of T & L*** | **p. 13** |
| 2a | The results show that Centers **provide inspiration** for a greater investment in EBIP’s. | A1, C1, E3, F3  B1, D2/ faculty and Center |
| 2b | Faculty describe an **increased understanding of student learning**, and the value of coming together to discuss teaching. | A1, C1, E3, F3  B1/ faculty and Center  D2 / faculty |
| 2c | Cross-case data show Center led initiatives collectively engaged faculty in a number of ways that **led to improvement** in their quality of teaching. | A1, C1  B1, D2, E3, F3/faculty and Center |
| 2d | **Faculty were committed** when the Center partnered with them. | A1, B1/ faculty and Center  C1, F3  E3 / faculty  D2 absent |
| 2e | Departmental **discussion of teaching led to curricular reform** that sparked on-going discussions and questions. | A1, B1, C1  D2/ Center  E3, F3/ faculty and Center |
| 2f | **Seed the use of EBIP’s through training programs** for peer mentors (e.g. Learning Assistants), graduate teaching assistants, and post-doctoral students. | A1, C1, E3  B1, D2 absent  F3/ faculty and Center |
| 2g | Faculty who utilized peer mentors felt the **training** students received **made the programs more impactful**, for both the mentors and the students. | A1, C1, E3  B1, F3/ absent  D2/ faculty and Center |
| 2h | Faculty who **used peer mentors were more committed** to the use of evidence-based practices. | A1, C1, E3  B1, D2, F3/ absent |
| 2i | Comments collectively reveal Centers are valued for the way they **promote the importance of teaching**. | A1 – F3 administrative level |
| 2j | Case data show that these Centers offer **support** to facilitate **the scaling of successful interventions**. | A1, C1, E3  B1, F3/ faculty and Center  D2 absent |
| 2k | Centers contribute to the institutional culture for teaching and learning is through the **leadership of affiliated faculty.** These disciplinary faculty, are engaged with the Center as research scholars or as partners in the use of EBIPs  e.g. Affiliated faculty led and supported departmental efforts to improve teaching by partnering with colleagues, while setting an example for the use of student centered practices. | A1 – F3  A1, B1, C1, E3  D2, F3/ faculty and Center |
| 2l | Centers played an **important role in disseminating** the results of research studies carried out at their local campuses  e.g. Local dissemination of studies often served to spark interdisciplinary conversations, which informed reform efforts in gateway courses.  e.g. Different modes and methods of dissemination were important to faculty engagement | A1 – F3 |
| 2m | Interview comments collectively show a **wide variety of faculty participation** in these seminars/institutes/workshops, ranging from senior to junior, tenured, as well as teaching track. | A1, B1, C1, F3/faculty and Center  D2/ faculty  E3 |
|  | ***Section 3: Broadening Participation and Institutional Capacity for STEM Learning*** | Pg. 16 |
| 3a | Center hosted events **bring together faculty, students, and upper administration**, in this way they increase awareness of grass-root efforts within departments. | A1/ faculty and Center  B1, C1, E3  D2/ faculty and Center  F3/ admin and Center |
| 3b  3c | The data show Center functions seek to **bridge the different disciplinary priorities** **by bringing faculty together to share common goals** and envision opportunities for synergies.  Cross-case data show Center **networks** to be an **important resource** through which they contributed to the expansion of institutional efforts. | A1, B1, C1, E3, F3  D2/ faculty and Center  A1, B1, C1, E3, F3  D2/faculty and Center |
| 3d | All Centers **hosted events to increase student awareness and interest** in STEM and related fields. | A1/ faculty and Center  B1, D2, E3, F3  C1 absent |
| 3e | Extensive engagement of Centers in areas that **provide authentic experiences for students**. | A1, C1 absent  B1/faculty and Center  D2, E3, F3 |
| 3f | Directors describe opportunities for **new mentors to be trained in strategies** that foster under-represented student success. | A1, C1, D2, E3, F3, /faculty and Center  B1 absent |
| 3g | The data in three cases also show that Centers **utilize their student network** to promote the benefits of these experiences to new students. | A1, B1, C1 absent  F3, E3, D2/ faculty and Center |
| 3h | The data show Centers support efforts directed toward **improved student engagement** through applied curricular experiences. | A1 absent  B1, C1, E3/ faculty and Center  D2, F3 |
| 3i | The data show faculty referenced these activities and programs, as evidence of existing campus efforts, allowing them to **submit more competitive proposals** for external funding. | A1, C1 absent  B1, F3/ faculty and admin  D2, E3/ faculty and Center |
| 3j | As faculty brought in more funding, contributions to broader impacts grew, which **increased support and opportunities for students.** | A1, C1 absent  D2, E3, F3/ faculty and Center  B1 faculty |
| 3k | K16 pipeline:  faculty describe engagement with their Center **to integrate innovative curriculum into the local high schools**.  **increased the visibility of their STEM programs**  these institutions are focused on expanding their student base through Center outreach. | A1, C1 absent  B1, D2, E3, F3/ faculty level  A1, C1 absent  B1, D2, E3, F3 / admin level |
| 3l | Center **functions that support improved teaching and learning are also perceived to broaden participation**. | A1, C1, E3, F3  B1/ admin and faculty  D2/ admin and Center |
