# Supplementary material for: STEM education centers: catalyzing the improvement of undergraduate STEM education
Source: Int J STEM Educ. 2018 Nov 12;5(1):47. doi: 10.1186/s40594-018-0143-2 (PMC6310466; doi:10.1186/s40594-018-0143-2)
Supplement: Supplementary file 2 — Center mission and upper administrator perspective. (DOCX 73 kb) [file 40594_2018_143_MOESM2_ESM.docx]

Additional file 2. Center mission and upper administrator perspective

| Institution | Mission | Administrative comments |
| --- | --- | --- |
|  | Source: Artifacts (web site, documents) | Source: Interviews |
| A1 | The mission of the center is to improve teaching and learning by integrating pedagogy and scholarship. This integrative approach promotes innovation in the classroom.  Programs and services focus on:   - Creating a shared teaching culture - Advancing the scholarship of teaching and learning - Classroom design to support innovative teaching   We work with faculty to design and evaluate evidence-based pedagogy. | “The Center, I think, is really helping some of our most vulnerable faculty feel empowered” (Vice Provost)  “I think that the Center does an outstanding job in trying to infuse the active learning strategies and evidence based practices into the classroom particularly in STEM.” (Undergraduate Dean)  “I think the Center has definitely had an impact, but I think it’s just at the beginning of, it’s maybe in the 3rd or 4th inning.” (Provost) |
| B1 | To improve teaching and learning in the STEM disciplines for students from grades K-16 through research and development. To bring together STEM faculty and faculty from other colleges to foster innovation, research, and intellectual exploration. To promote ideas and projects that will make a difference in the teaching and learning for all types of STEM learners. | “The Center carries out valuable research, which informs the campus and the broader community at large. (Center name) is internationally known for the high quality research it carries out. I have been thinking also that I’d like to run our Bridge programs through (Center name) this would serve us well.” (Provost)  “The Center has allowed us to make massive changes. It sets up the environment, and there is recognition that ‘This is important,’ by the faculty. It has allowed us to attract strong leaders within the STEM disciplines to lead departmental efforts.” (Dean of College of Natural Science) |
| C1 | To promote excellence in teaching and learning in the sciences. We bring faculty, post-doctoral scholars, and graduate students together to discuss undergraduate education and share innovations. Our programs and services promote and encourage departmental coordination. | “The Center was a way to expand both the resource base and the inspiration for more investment in thinking about kind of emerging ... I mean we weren’t calling it active learning. The range of kind of evidence-based things that we have now- the Center helped us to catalyze that.” (Assoc. Dean of College of Science)  “it also helps faculty to write proposals, which lead to broader impact. So it really impacts the college in multiple ways, and it also acts as a great resource, at the university level.” (Dean of College of Science)  “Another thing that the Center has done. We have almost 90 full time professional track faculty in our college at this point. And it’s taken some of the people who are an n of one in a small department. And it’s starting to give them a community of people who feel the same way about the importance of teaching, want to be better, want to develop new ideas, want to engage with new ideas. That we can foster this better across the departments than some who are isolated may think.” (Assoc. Dean of College of Science) |
| D2 | To provide coordination and support for educational initiatives in STEM   - Identify grant opportunities and coordinate interdisciplinary participation in pursuing them - Identify research opportunities for undergraduates   To serve as an umbrella organization to facilitate and promote STEM education initiatives.  We help to connect faculty w/university resources which guide the reform of classroom instruction.  To support and foster collaboration among Arts and Science, engineering, and education.  To develop and implement PD for K-12 teachers locally and regionally. | “The Center made STEM more visible to faculty and administrators, and importantly to our students too. The Center attracted students to STEM through increasing job awareness, something we had not done before on this campus. And I should say that we have a very high, in fact most of our students are 1st generation and work part or full time. This is a surprisingly high number roughly 85% to put that in full perspective.” (Dean of Natural Science)  “We got behind the Center. Our goal was to encourage some of our STEM faculty to engage in STEM education research, and (director’s name) is a good example of someone who is very interested.” (Vice President for Research) |
| E3 | To improve STEM education and outreach initiatives. We foster collaboration between STEM educators and education researchers.  To foster educational transformation, by carrying out scholarship of teaching (DBER, SoTL). Our programs promote diversity in STEM for underrepresented student groups. | “We want to bring these efforts together and give STEM education the importance that it deserves. Every time you create some kind of a structure and you give it a name and you put everybody under that umbrella, then all of a sudden - it’s known that it exists.” (Dean of College of Science)  “Yes, so (Center name) assists us in ways that help the faculty to adopt teaching strategies that improve student success and therefore retention.” (Dean of College of Science)  “The other thing the Center has done, is they laid out an institutional strategy for STEM education, research support. And so you look at what the funding opportunities are and you can probably name ... There’s like 10 federal programs that support this kind of thing and they laid out a strategy that’s like “Okay, these are the 10 that if (institution’s name) had one in each one of these programs, we would be top tier.” Okay, what are the deadlines for each one of those? And who would be potential PIs that could go after each one of these? They helped to start to build a larger effort around these, and it’s incredible how that strategy has come to fruition.” (Vice President for Research) |
| F3 | Our center fosters a culture of excellence in STEM which is inclusive of underrepresented groups. We seek to nurture diversity by (a) actively supporting and advocating for student and faculty success in and out of the classroom, (b) catalyzing educational research in STEM to improve student learning, and (c) building communication between the University and external stake holders which leads to collaborative efforts to improve STEM education and opportunities. | “(Center’s name) has the very important function of funding undergraduate research and mentoring for students. It also provides data to the university regarding STEM. (Director’s name) has an expansive vision of STEM and is focused on being inclusive. (Center’s name) cultivates research activity, and diversity for all STEM disciplines.” (Vice Provost)  “I see it as a mechanism to enhance undergraduate research at this university, and contribute to student progression and learning, and so forth. I think, the structure allows us a fast lane approach to be able to take those kind of issues on and move along.” (VPR) |
