# Supplementary material for: STEM education centers: catalyzing the improvement of undergraduate STEM education
Source: Int J STEM Educ. 2018 Nov 12;5(1):47. doi: 10.1186/s40594-018-0143-2 (PMC6310466; doi:10.1186/s40594-018-0143-2)
Supplement: Supplementary file 3 — Cross-institutional examples of the ways in which centers engage in educational research. (DOCX 74 kb) [file 40594_2018_143_MOESM3_ESM.docx]

Additional file 3. Cross-institutional examples of the ways in which centers engage in educational research

Educational research includes assessment and evaluation linked to educational research carried out by center.

| Improving STEM learning through educational research | | |
| --- | --- | --- |
| Center | Department | Upper Admin |
| Self-described role | Engagement of faculty and chairs | Center contribution |
| Institution A1 | | |
| “We support faculty and we do know how to get faculty to try evidence based practices, and this leads into evaluation, because you cannot expect most faculty to try them unless you help evaluate them right?  We believe and the university believes, that it’s something that we should be studying to see whether it’s effective to spread over the university or to send out into the education world.” (Director) | “to sit there for two and a half days and have them put up - this is what the research is showing us and it’s black and white, what the research is showing us. So getting faculty to do things like that, and I don’t think it would work in an hour. I had to be immersed in it from different dimensions and see that there’s many different techniques. Everything that they present is based in research-based findings that this is effective or can be effective.” (STEM faculty)  “I teach 2 undergraduate courses. Both have benefited from HHMI funding. The (Center’s name) was involved in helping us with some of the assessment of the changes that we made to the courses.” (STEM faculty) | “We just got done studying that and we found some really interesting results, and the Center is of course taking all of this back, communicating, and working with the faculty. As I understand this engages faculty. They then work with (Center name) to make changes.” (Undergraduate Dean of Science) |
| Institution B1 | | |
| “Here at (Center’s name) we have more than 15 externally funded grant projects underway. They are curriculum and instruction innovation and development grants.” (Director of Communications) | “What’s a meaningful measure of assessment when you’re doing something new from the ground up? That’s where we’ve engaged with (Center name). They advise us in this area.” (STEM Dept. Chair) | “Much of the institute’s research supports the k-12 pipeline and that is always useful, given that we draw 75% of our students from in-state. However, it has several grants that contribute to our undergraduate efforts and it brings together our faculty engaged in (name of national STEM initiative) where faculty are engaged in improving our gateway courses.” (Provost) |
| Institution C1 | | |
| “It’s so important at a research university you need to talk the same language. You need to talk research. You can’t just do things. So if I would just give the service that I’m giving here, without the research, I think that it would lower everything.”  (Director)  “It’s always been very natural for us to carry out research as part of our work, and we actually use it as the hook to engage our faculty, because they see everything as a research problem if you let them.” (Assoc Director) | “Yeah, so it took a long time, then we started generating data and none of us have any educational data expertise and that is really when (director’s name) joined the group, because we needed someone from the science education side to sort through all the data.” (STEM Faculty engaged in curricular reform)  “And the integral part was having (director and assoc. director’s names) doing some of the assessment and just that whole side of it was completely new to me, just seeing the whole development of the concept of inventory was all new. I knew that I may never be at the level of say Mike where that is his full-time job, I have to run a research lab, I have to get grants, I have to do this stuff. But there was always an incentive to come, because it made my teaching easier. And I knew I could do things that would make it easier, meaning if they understand it more, you have less frustrated kids showing up in your office going, ‘I do not understand.’” (STEM faculty)  “We’ve gone to them for assessment. In the sense that we’re doing something and we want to see how effective it is. Which, yes, that’s part of learning outcomes assessment. We’ve gone to them for specific assessments, specific courses.” (Math Dept Chair) | “We have had a run of HHMI education grants—and you might be familiar with that program. It’s kind of evolved over time. And I would say at the earliest stages, they really had two interests; individually mentored research for talented students, and curriculum development. The Center has engaged in research to help us understand the impact of these efforts.” (Dean, College of Science) |
|  |  | “There are also some chairs now that have been very engaged with these efforts. It’s like Steve in biology has been a first PI and a co-PI on two teaching & learning grants, and he helped foundationally to get this initiative off the ground. He’s the (STEM department’s name) chair and has a quantitative background.” (Associate Dean, College of Science) |
| Institution D2 | | |
| “We gathered the data for our (NSF grant name) and students who participated in some type of networking events had significant improvement in retention and graduation in STEM, and these were students who may have otherwise not completed their degree.” (Director) | “Some of us who are interested in the education side of things were more engaged with the Center. We were interested in ways to engage our students so they would be more successful.” (STEM Faculty) | “The Center really brought faculty together around externally funded awards. They all contribute, but when the Center was more active they created community for those faculty interested in research related to their classroom teaching.” (Dean of College of Science) |
| Institution E3 | | |
| “I think that if (Center name) becomes too service oriented, we will lose our focus, which needs to be scholarship in DBER. And the reason (Center name) needs to be scholarship is, it is through external grants that we get our money. So, I’m very hesitant to allow it to become an administrative entity that relies on internal funds, because we lose the rigor and focus of disciplinary scholarship.” (Director) | “We try to assess what is the impact, so when you have LA’s versus not have LA’s in the classrooms how do the students do?(Center name) helps us evaluate this. They have peer interaction, peer mentoring that goes on, and we’re getting some mixed results, I guess. Generally positive, but there are some pieces of the data where we go, Huh, I wonder what happened there.” (STEM Dept chair) | “So we had two faculty members get tenure recently, because of the work they were doing for the Center. So now that validates the work that is done in the Center. It is valued by the institution, because it’s real research.” (Dean, College of Science) |
| Institution F3 | | |
| “The other thing is…is that my root to the faculty is through their research. How they write their educational plans. How they evaluate outcomes. How they do their broader impact, so wearing their research hat.” (Director) | “I’m a PI on (NSF grant name). In fact, (grant name) ... (Center director’s name) was our external evaluator on (grant name).  (Center director’s name) has always been available as a person that we would discuss things with. ‘What are we doing here? What are we learning? What should we do next? How do we know if this is valuable?’ These are the kinds of conversations that everybody on staff at (NSF name) has had with (director’s name) over the years.” (STEM Faculty) | “(Center name) provides data to the university about undergraduate STEM, so that we can make informed decisions. (Center name) also provides students with funding for undergraduate research and mentoring.” (Provost)  “We really need the data analytics and that’s a really key role of the Center.” (Vice Provost) |
