# Supplementary material for: STEM education centers: catalyzing the improvement of undergraduate STEM education
Source: Int J STEM Educ. 2018 Nov 12;5(1):47. doi: 10.1186/s40594-018-0143-2 (PMC6310466; doi:10.1186/s40594-018-0143-2)
Supplement: Supplementary file 4 — Cross-institutional examples of the ways centers enhance the quality of teaching and learning. (DOCX 73 kb) [file 40594_2018_143_MOESM4_ESM.docx]

Additional file 4. Cross-institutional examples of the ways centers enhance the quality of teaching and learning

Quotes are representative examples and thus represent interview comments that were expressed by three or more participants within each level

| Enhancing the quality of teaching and learning | | |
| --- | --- | --- |
| Center | Department | Upper Admin |
| Self-described functions | Engagement of faculty and chairs | Center contribution |
| Institution A1 | | |
| “Much of our time is spent working with faculty to practice and implement EBIPs and innovations. We have several departments engaged to different degrees, and it is slowly expanding. It is interesting and takes a slightly different form in different departments.” (Center Director) | “Yeah. I think the (Center’s name) plays an important role in this. They try to create a culture where teaching is valued.” (STEM faculty) | “(Center name), I think, is really helping some of our most vulnerable minority faculty feel empowered providing them with a key resource.” (Vice Provost)  “The Center provides a lot of workshops and great support for faculty who want to do a better job teaching. We get great reviews on the workshops and things that they do.” (Provost) |
| Institution B1 | | |
| “Our having these little mini seed grants is a way to bring in potential interactions with professors. What (Center’s name) can do is to connect some of these disciplinary experts with each other not necessarily in an institutional way. That’s the unique role that (Center’s name) can play. The Center provides a space where those interested in educational research can come together.” (Director) | “Now (Center’s name) is where we meet. Having this place and the support of (Center’s name) has enabled us to have these conversations, basically, which have, I think, been the mainstay of this project.” (STEM Faculty)  “We may come here and interact with other researchers and talk about, ‘How are you assessing this, how are you doing that?’ Really, I see (the Center) as kind of a hub for us all to come together and make sure that we are aware of the research that’s going on.” (STEM Faculty) | “We rely on them (the Center) and the people who participate in (Center’s name) to make sure we have a strong scholarly base for what’s presented in the principals of teaching workshops. To me, the crucial element there is to pay attention to learning.” (Dean of Undergraduate Education, STEM faculty) |
| Institution C1 | | |
| “we needed something close to home staffed by people who would be interacting with our faculty on a day-to-day basis and providing programming that our faculty would understand from the very beginning was directly relevant to their everyday teaching responsibilities. It’s, ‘Oh, I see my colleague who’s teaching the same class as me doing X. I can do X, too,’ so we tried to keep it very, very close to home, lots of interaction with faculty and administrators within the college, and also structured in a very familiar way like a scientific research group.” (Director) | “Not only did I take things away that Brian and I had started introducing in class—we started clicker questions in the class, started active learning such as we had today in the class. Not only did I do all that, but also my reviews went up. Students liked the class, they liked the way I taught it more. I went from doing okay, I was never I’d say a bad teacher, to doing much better. In the end we stayed with it because we think it does work. Not only for the students, but also for us.” (STEM Faculty) | “There’s a real culture of respecting the teaching faculty, the Center promotes this, and we freely understand that they’re every bit as important as anyone else, if not more so. And so the idea and the philosophy is that people have to be excellent at what they do, and really, everyone has huge value and a fantastic teacher is no less important than a fantastic researcher.” (Dean of COS) |
| Institution D2 | | |
| “We support student success and retention by linking them with opportunities for research and internships, these connect them with faculty outside of the classroom. Students have told us that these made their classroom experiences more meaningful, because they cared more.” | “The Center supports us with seminars, these used to be monthly, now they are a couple of times a semester and they are well attended. These really help us to see what we can do. Also these are some of the only times I see my colleagues in other disciplines…” (STEM faculty) | “With (Center director’s name) help we were successful in engaging most of the college of Natural Science departments and had representation from Engineering. Faculty from these departments were engaging in undergraduate research and STEM ed research providing increased opportunities for students.” (VPR) |
| Institution E3 | | |
| “Initially the Center was really driven by the education research group. It has expanded to include a journal club, and programs to improve student success, like LA and xxx, which are all grant funded.” (Director)  “The focus of this (Center’s name) initiative shifted to how do we help faculty transform their classes and get more students through? Retention and on time graduation, and that then became the mechanism by which we are now I will say on the cusp of institutionalization.” (Director) | “(SEC name) does focus on improving teaching and transforming classroom practice. The DBER part is one little prong of (Center name) but then there’s a larger effort and a more inclusive effort within (Center name), which is really about transforming STEM teaching.” (STEM faculty) | “It is taking a multi section course and using faculty member ... I think it’s faculty member and a student. The third member of that team would be I think a teaching assistant maybe, and they’re going to try to take approaches to learning that have been successful, transformed learning in one section that a faculty member who’s been associated with (Center name) has experienced, and try to then expand it to other sections of the same course taught by other faculty.” (Vice Provost for Academic Affairs) |
| Institution F3 | | |
| “Some of my workshops have been held at the Center for Teaching and Learning. So, any of the faculty that might want to come and hear some mentoring strategies, especially for underrepresented students. And then, (associate director of CTL) & I’ve worked with on a couple of occasions to just help support what I’m designing and creating for faculty who are going to be working with minority students.” (Director)  “I can help show the data and point and give the stories of where some faculty development might be some hot spots that I can assist with or we can get other students engaged to get the student voice.” (Director) | “We are engaged in evidence-based learning practices with (Center’s name). Looking at how to bring this into our courses and curriculum. The Center initiates and engages us in discussion, and this is definitely of benefit to our students, because the paths are made clear.” (STEM faculty) | “We have a project, you’ve heard about, the (NSF grant name), our second (same NSF grant name) grant, propagating evidence-based best practices. That was a long effort over a couple of years that started with (STEM Center’s name) and the Center for Teaching and Learning working together to start developing workshops for faculty on evidence-based instructional practices and STEM teaching. Then we moved into action research, and then education research, there are faculty learning communities. There was just a whole range of programs.” (VPR) |
