# Supplementary material for: STEM education centers: catalyzing the improvement of undergraduate STEM education
Source: Int J STEM Educ. 2018 Nov 12;5(1):47. doi: 10.1186/s40594-018-0143-2 (PMC6310466; doi:10.1186/s40594-018-0143-2)
Supplement: Supplementary file 5 — Cross-institutional examples of center functions which broaden participation and institutional capacity in STEM learning. (DOCX 74 kb) [file 40594_2018_143_MOESM5_ESM.docx]

Additional file 5. Cross-institutional examples of center functions which broaden participation and institutional capacity in STEM learning

| Broadening participation and institutional capacity | | |
| --- | --- | --- |
| Center | Department | Upper Admin |
| Self-described functions | Engagement of faculty and chairs | Value of center functions |
| Institution A1 | | |
| “I think too, that the number of workshops that we do, that are more directly and consciously on inclusive teaching topics, like stereotype threat, growth mindset, and understanding bias. Even in a STEM Institute where faculty came to maybe learn about how to teach students how to critically read scientific literature, you are going to get these other things mixed in with it.” (Center staff) | “We’ve teamed up on a couple of other things. There’s an interest in increasing diversity in STEM. They’ve brought in a professor in math from the University of Xxx.” (STEM Faculty)  “Our students have more varied abilities now… The Center initiates discussion around topics related to teaching, and the impact on student learning. It also provides a meeting place for us to gather to continue these conversations.” (STEM Faculty) | “Yeah, I believe that this particular issue is in the 5 most important things we are facing because we are graduating low income and underrepresented students. Their graduation rate is no different than the wealthy students who are full pay, but what we are not doing is giving everybody the same fair chance to be a scientist. Part of the reason is because the methods that we use to teach—writing on the chalkboard and all that stuff is a 100 years old if not more. The Center is supporting our mission to improve in this area.” (Provost) |
| Institution B1 | | |
| “And yes once we innovate, once we create new materials and new instructional practices, we have to figure out how to get them out there. IN that sense there is very much a secondary kind of focus on outreach whether its professional development whether it’s materials dissemination. We want to get our good work out there.” (Assoc. Director) | “We are working with (Center name) to introduce the class into high school. We’re intending on going for a grant too, it’s a STEM-plus C grants, because one of the main parts of the practice is computation, so we do a lot of computation in the class. Since that has become an importance in high school as well, we’re looking to get it funded to bring the course to (state name) high schools.” (STEM Faculty) | “The biggest single thing that happened in the last year is two weeks ago we got a grant from the Gilbert foundation to build a (very large urban city/high poverty/large urm population) scholars program. The Center is part of this. We’ll be working with high schools in (urban city name), and we’re starting to figure out, I’m talking with people in the college of education who have connections (urban city name) high schools.” (Dean of Undergraduate Education)  “Support for k-12 helps the pipeline and that is always useful! 75% of our students are in-state.” (Provost) |
| Institution C1 | | |
| “We have a lot of experience working with chemists as well, because the old college is biology and chemistry. But we’re brand new working with computer science and physics and math. And we’re learning from their students and also from our interactions with faculty that the cultures are quite different. It would help a lot to have more expertise coming from the discipline. And especially since the case that we’ve made all along is knowledge of the discipline is critically important. You can’t be a generalist and really do the kinds of things that we’re aiming to do. It gets you so far, but it doesn’t get you everywhere you want to go.” (Director) | “Something that had always been suspected but we never really had the data within our group was that, students who came into (state name) as incoming freshmen consistently did better across the curriculum than students who came in as transfer students from the community colleges and so we actually, as part of the grant we set up a relationship with one of the major community colleges, (xxx) College, and had one of our Microbiology faculty members there as part of the learning community for a year or two. The Center helped us to set this up. The idea was to make the introductory microbiology course more comparable, to bring the same case studies, to bring the same curriculum back and forth so that when students did transfer in, that they would be able to come in more seamlessly.” (STEM faculty) | “I think one of the big challenges is the range of abilities of our students. Students in our introductory courses, tend to be our weaker students… and they don’t know why they’re there. But I think the whole active engagement in transforming pedagogies, both on campus and in the college, is a big part of addressing this, helping those students to succeed. (Center directors name) works closely with faculty to innovate.” (Undergraduate Program Director) |
| Institution D2 | | |
| “One of the primary roles of our Center is to equitably link students to undergraduate research opportunities. We send out announcements via email and social media, as well as through faculty, advisors, and student groups. We also host student gatherings for everyone to hear about what a great experience it is. For some we also provide the necessary funding. Some of that of course is through LSAMP.” (Associate director)  “Many of our programs require faculty to have training prior to becoming an undergraduate mentor. HHMI for example. We provide that, and we help match students to mentors.” | “The STEP grant provided increased funding to create summer bridge programs. We run those through the Center, and the Center has helped us organize those. Along with networking opportunities for new students to meet upper level STEM students. These have helped new students feel connected.” (STEM Faculty) | “The Center hosts events for K12 teachers and students throughout (name of state region). Faculty engage as part of their outreach and Broader Impacts. These are important to engage our local commuter population.” (Dean, College of Science)  “We have a large population of commuter students who have jobs. Most of which are first generation… about 85% of our students actually, so we put significant effort into K12 and local community outreach. The Center plays an important role here. It helps to coordinate these opportunities and importantly it facilitates communication between schools and the university.” (VPR) |
| Institution E3 | | |
| “Yes, so the Center works on inclusion and diversity through its funded programs. I’m all about pressure. I have told the Dean, I’m happy to work with departments, where all of a sudden say the biology department goes from how are my student violations… to asking how about students learn and what’s your evidence that they learn. That’s a very uncomfortable question for many faculty and I’m perfectly happy to have uncomfortable questions directed toward them.” (Director) | “if you look at what we’re trying to do in terms of our mission statement and our core values and strategic plan, career preparation is elevated so high. There is so much natural synergy between how we talk about career preparation in a kind of comprehensive expansive way. We want to integrate that into our efforts toward course transformation.” (STEM Faculty) | “We’re focused on, obviously, diversifying our student base. We are very interested in pulling a lot of underrepresented students from the xxx City School District, for example. More importantly, nationally we know that the country is going to go through this radical diversification transformation, and we need to be prepared as a STEM-oriented institution to really educate those students that are going to be increasing in numbers. That’s really deliberate on our part. We need to make sure that our pedagogy really supports their learning and that’s a direct contribution from (Center name and Center director’s name).” (Provost) |
| Institution F3 | | |
| “For the STEM Diversity piece I’m really providing support from recruitment through graduation to underrepresented students. I also, through the LSAMP piece, work with community college students as they transfer, specifically at the College of Western xxx. And, I would say probably that the main support for underrepresented students looks like connecting them to apply for learning opportunities. So, whether that’s undergraduate research positions, internships, professional opportunities, those kinds of things, to help clarify kind of what they want to do with their careers in STEM and help them engage at the university.” (Center Staff) | “Another connection point, we have an NSF (grant name) right now. The Center director is the co-PI, she was a big part on bringing that grant in. Its called (name). Aligning XXX and structures to enable xxxx. We just got back from a retreat that had faculty from ecology, EUB, and faculty from geo sciences, human environment systems, anthropology and so forth. We started chatting about some of the barriers for them working together and moving forward. We’re collaborating with a group of about 11 faculty right now with an extension of larger circle of about 25.” (STEM Faculty) | “When we’re going to do a new outreach activity. So, last year we started an all-girls first robotics team. Actually, I was trying not to take on another project, but it escaped me. I knew (Center staff member) was interested, I spoke with her and she took charge of it. But we hosted it in the College of Engineering and a couple of my staff are mentors.” (Dean of Engineering) |
